# Modelling and metrics for optimal sizing of renewable power plants supplying green hydrogen generation systems

**DOI:** 10.1038/s41598-026-36987-0

**Published:** 2026-01-26

**Authors:** Mobin Naderi, David A. Stone, Erica E. F. Ballantyne

**Affiliations:** 1 School of Electrical and Electronic Engineering, University of Sheffield, Sheffield, UK; 2 Sheffield University Management School, University of Sheffield, Sheffield, UK

**Keywords:** Battery energy storage systems, Green hydrogen, Modular modelling, Renewable energy, Sizing problem, Energy science and technology, Engineering

## Abstract

This paper presents a modular modelling approach for long-term analysis and design of renewable-powered hydrogen generation and storage facilities, encompassing both power generation and hydrogen system components. The proposed model can be used to integrate different sizes of solar and wind energy resources, different battery energy storage systems, a backup power source (if required), and main hydrogen system modules in power demand calculations. As a part of the paper’s novelty, the proposed modelling approach is modular and case study-free, which allows for generalisation to a variety of case studies. The expandability of the modelling method is strengthened by presenting a unified modelling framework for all modules required in modelling the system. As the second main paper’s contribution, a comprehensive set of performance metrics is proposed to support a multi-objective optimisation framework for optimal sizing of system components. Although the metrics focus on different technical and economic aspects, environmental issues can be covered using some metrics, like the grid share of total energy requirements for the hydrogen system. Both proposed modelling and sizing methods enable renewable power plant designers to evaluate different configurations and make informed decisions based on weighted performance criteria. The proposed model and sizing problem are implemented in a combined Editor and Simulink environment in MATLAB for a case study as a real feasibility study in the UK to operate a renewable-supplied hydrogen system, including a 1 MW electrolyser. Simulation results for the representative case study validate the model’s behaviour and its reliability through various primary output profiles, e.g., power profiles, and secondary outputs, e.g., met hydrogen demand and levelised cost of hydrogen. The proposed modelling and optimisation methods can easily be expanded for case studies with more technical data or different load demands, e.g., combined hydrogen, heat, and power.

## Introduction

### Problem definition

Hydrogen is of interest in a range of sectors, including heavy industry and long-distance transport, as an energy carrier as well as an energy storage technology with many advantages^1^, e.g., a considerable energy density of 39 kWh/kg against 12.7 kWh/kg for diesel fuel and 0.15 kWh/kg for a lithium-ion battery^2^. For example, the UK government has reported key policies for the low-carbon hydrogen economy and a forward scoping of upcoming opportunities^3^ as well as hydrogen transportation and storage infrastructure requirements^4^ to pave the way for new hydrogen industries covering a wide range from huge grid-connected storage systems^5^ to infrastructures of hydrogen electric vehicles^6^. Large grid-connected hydrogen generation/storage stations can be used for managing a considerable amount of wind energy curtailment in the UK and Nordic countries to generate green hydrogen^7^. On the other hand, hydrogen can be used in remote areas and off-grid microgrids as inter-seasonal long-term storage. Moreover, some industries intend to attend the future hydrogen market through systematic generation and storage of green hydrogen from solar and wind energy resources.

In the case of industries intending to generate green hydrogen, a feasibility study and optimal sizing of the main modules of the power plant is necessary, generally including renewable energy sources (RES), energy storage systems, and at least a backup resource to cope with a mandatory hydrogen demand. A basic step for optimal sizing is techno-economic modelling. In terms of technical modelling, since both energy and power metrics are challenging in RES-included feasibility studies and are affected by seasonal weather changes, long-term detailed modelling according to power profiles is required. On the other hand, different metrics can be used to explore various aspects of the sizing problem, including technical, economic, and techno-economic performance indicators, and finally, environmental metrics.

### Literature review

Table 1 presents a review of the most relevant literature to this paper, focusing on the idea of optimised renewable utilisation for large-scale hydrogen production. Important features are categorised, including sizing and modelling methods, the level of used and/or presented details in modelling, metrics used in the analysis, and the study category of the literature. Some of these features are adopted from a recent review paper on long-term assessments of green hydrogen systems^8^.

A hybrid energy system including all basic modules, i.e., RES, a (hydrogen) storage system, and a (diesel generator) backup to supply a health care centre is optimally sized using several optimisation tools and sorting methods, e.g., non-dominated sorting genetic algorithm (NSGA) and analytic hierarchy process (AHP)^9^. The authors have presented a detailed model of the optimisation problem, including both technical and economic features. In^10^, the authors have maximised the profitability of hydrogen production from wind and solar by optimising the electrolyser size. In^11^, HOMER is used to solve another single-optimisation problem by applying a sensitivity analysis on the levelised cost of hydrogen (LCOH). HOMER-based modelling and optimal sizing based on net present cost (NPC) are used in more papers to design an off-grid microgrid powering a hydrogen and electric vehicle charging station^12^, a set of hydrogen stations powered by wind and solar RESs considering some artificial intelligence and metaheuristic algorithms for dynamic pricing^13^, and a solar-supplied hydrogen generation system^14^. In contrast^15^, uses a detailed techno-economic MATLAB model for feasibility analysis of a certain small-scale wind-solar-hydrogen plant without any optimal sizing but considering reasonable sizing rules for a required hydrogen demand. Several economic metrics are used in feasibility analysis, including LCOH, levelised cost of energy (LCOE), NPC, and internal rate of return.

In^16^, the authors introduce different RES-based power plant configurations for power-to-hydrogen systems and employs a multi-objective sensitivity analysis to find optimal source-per-demand size ratios. Although the authors’ effort to present a general methodology using several normalisations is appreciated, the analysis results still depend on the RES installation location. This fact is highlighted in^17^, where a part-load operation of the hydrogen system is investigated due to the volatility of RES. Despite the lack of an optimisation method, data-based modelling and sensitivity analysis on several techno-economic performance indicators present analytical results to find the best sizes of RES and electrolyser. Another paper has focused on weather data uncertainties to find a robust solution only in RES sizing for a hydrogen refuelling station. Carbon emission is one of the metrics used in decision-making^18^. However, battery and backup systems are not considered in this study. In^19^, another large-scale hydrogen production system is studied for high-potential wind and solar energy areas in Morocco, considering a grid connection to sell surplus energy. Although the model seems comprehensive, the detailed module modelling is not presented, as there is no optimisation for sizing the system.

In^20^, a detailed modelling in MATLAB/Simulink is presented for RES and hydrogen tank sizing for generating green hydrogen, but a special case study of utilising surplus energy from a hydro power plant and a solar farm is investigated. There is no optimisation, and decision-making for sizing is only based on graphs of a set of metrics, excluding battery and backup metrics. In^21^, the same graph-based decision-making method is used for sizing a solar-battery-hydrogen system, which is a more generalised study system, but the proposed modelling method lacks details to be generalised for other systems. The authors in^22^ have used a wide range of sensitivity analyses using several technical and economic metrics for different configurations of solar, wind, battery, and electrolyser. Nevertheless, some changeable parameters or their ranges do not seem to be applicable, e.g., electrolyser efficiency changes between 60% and 100%.

**Table 1:** Literature review for sizing and modelling hydrogen generation systems powered by renewables.

Ref.	Main idea	Sizing method	Modelling method	Level of presented details in modelling	Metrics used in analysis	Study category	Main shortcomings compensated in this paper
^9^	Sizing using several optimisation tools and sorting methods	Multi-objective optimisation	Detailed long-term techno-economic	High	NPC, unmet demand	A hybrid energy system with a certain demand	Case study-based presentation of modelling and optimisation
^10^	Electrolyser sizing to maximise profitability	Single-objective optimisation	Mostly economic	Very low	Total profit	Grid-scale green H_2_ generation	Low attention to technical constraints and metrics Brief methodology presentation
^11^	Green H_2_ refuelling station	Single-objective	HOMER-based techno-economic	Medium	H_2_ production cost	Case study-based HOMER modelling and sizing	Low flexibility of HOMER model to consider real multi-criteria grid electricity pricing (except^13^) Lack of detailed degradation modelling Simplified long-term models of modules
^12^	H_2_ and electric vehicle charging station	Single-objective			NPC		
^13^	AI-based dynamic pricing in optimised sizing	Multi-objective			NPC, LCOE, LCOH		
^14^	Large-scale solar-powered H_2_ system	Single-objective			NPC, LCOH		
^15^	feasibility analysis for a renewable power plant generating H_2_	Multi-objective economic analysis	Detailed long-term economic	Medium	NPC, LCOE, LCOH, cash flow, IRR	Small-scale H_2_ storage	Lack of technical modelling details and focusing on economic modelling and metrics
^16^	Optimal sizing via source/demand component size ratios	Multi-objective optimisation	Detailed long-term techno-economic	Medium	LCOH, UF	Different power to H_2_ configurations and their optimal sizing	Lack of detailed and generalised module modelling Lack of battery efficiency metrics
^17^	Data-based electrolyser sizing (using surveys)	Multi-objective SA	Data-based techno-economic	Low	LCOH, CAPEX, UF	Potential green hydrogen production locations	Extremely case study-based Lack of detailed modelling
^18^	Optimal sizing considering uncertainties	Single-objective optimisation	Detailed techno-economic	High	NPC, LCOE, carbon emission	Very large-scale green H_2_ production	Lack of battery and backup models and metrics in the system
^19^	RES technical sizing for green H_2_	Multi-objective SA	Technical	Low	Energy and H_2_ production	Very large-scale green H_2_ production	Lack of detailed and generalised modelling Lack of economic modelling and studies
^20^	Using surplus energy of a hydro power plant	Multi-objective SA	Data-based techno-economic	Low	Income, H_2_ production, UF	large-scale green H_2_ production	Lack of optimisation Lack of battery and backup models
^21^	Balancing Electrolyser Sizing and Storage system	Multi-objective SA	Specification-based techno-economic	Low	NPV, LCOH, UF	green H_2_ production from solar energy	Lack of detailed modelling Lack of optimisation
^22^	SA for solar and wind plants	Multi-objective SA	Specification-based techno-economic	Medium	LCOH, LCOE, operation time	green H_2_ production from solar and wind curtailments	Lack of grid/battery models and metrics Assuming only excess energy from RES
^23^	Optimal sizing and reliability analysis via PSO and SA	Single-objective optimisation	Detailed techno-economic	Medium	LCOE, ENS, LOLE	Off-grid hydrogen generation	Lack of battery modelling, metrics, and sizing

*AI* Artificial intelligence, *CAPEX* capital expenses, *ENS* energy not supplied, *H*_2_ hydrogen, *LCOE* levelised cost of energy, *LCOH* levelised cost of hydrogen, *LOLE* Loss of load expectancy, *NPC* net present cost, *IRR* internal rate of return, *SA* sensitivity analysis, *UF* utilisation factor.

### Paper contributions

Here, this paper concentrates on detailed long-term modelling, which has received less attention in the literature, and proposes a multi-objective optimal sizing of a renewable power plant powering a hydrogen production and storage system as a complete hybrid energy system including RES, an energy storage system, and a grid connection used as a backup. The last column of Table 1 presents the main shortcomings of the reviewed literature that are addressed in this paper. Moreover, Table 2 clarifies concrete advances achieved in this paper over prior works. Accordingly, the main contributions compared to the existing literature are listed below.

- The paper presents a unified modelling framework for detailed long-term modelling of all modules used in the RES-powered green hydrogen production and storage system.
- The proposed modelling method is modular and free of case study data, which can be easily generalised for a large range of case studies, including main modules modelled in detail. Although modelling according to exact measured data has some advantages^17^, this data is not always available. That is why the proposed modelling method is based on manufacturer data sheets, which have a higher degree of availability.
- To support modelling electrical power and hydrogen mass rate in an hourly resolution as basic modelled signals, the details of module modelling, including the power part and logic control, are precisely presented, unlike existing relevant papers^10,11,13,16^, and^17^. Note that realising these signals makes the model capable of modelling energy/hydrogen storage state-of-charge (SOC) and corresponding degradation processes, as well as considering detailed logics required for a precise operation of the power plant and the hydrogen production station, suitable for precise long-term sizing studies.
- A set of comprehensive techno-economic metrics is proposed to consider different aspects of the sizing problem. In addition to well-known and necessary metrics like met hydrogen demand (MHD), known also as utilisation factor, LCOH, and capital expenses (CAPEX), new efficiency metrics to consider RES spillage, battery energy storage performance, and backup source performance in optimisation are proposed, which have not been introduced earlier to the best of the authors’ knowledge. To take all these important technical and economic metrics into account, a simple normalisation-oriented multi-objective optimisation method is presented, considering weighting coefficients to find the sizes of the renewable power plant.

**Table 2:** Comparing the paper and the most related literature in terms of the main paper contributions.

	Unified modelling framework	Case study free (generalizable) modelling method	Detailed techno-economic modelling	Proposed efficiency metrics^a^
^9^	×	×	✓	×
^10,17,19,21^	×	×	×	×
^11-14^	✓	×	×	×
^15^	×	✓	×	×
^16^	×	✓	×	✓^b^
^18,22,23^	×	✓	✓	×
^20^	×	✓	×	×
^24^ ^c^	✓	✓	✓	×
This paper	✓	✓	✓	✓

^a^These efficiency metrics are to consider RES spillage, battery energy storage performance, and backup source performance in the optimal sizing problem.

^b^Except battery storage performance metric

^c^This paper does not deal with a specific use of RES for hydrogen production and storage systems, but it is highly related to RES-based hybrid energy system modelling.

### Paper organisation

The rest of the paper is organised as follows: section "Proposed long-term modular modelling method" presents the proposed long-term modular modelling method. The section "Economic modelling" provides economic modelling. The comprehensive set of metrics for the optimal sizing problem is proposed in the section "Proposed comprehensive set of metrics for the optimal sizing problem". The section "Proposed methodology for sizing problem" deals with the sizing problem methodology. The section "Case study and results analysis" investigates a case study and the corresponding results. The section "Conclusion" concludes the paper.

From a high-level modelling and optimisation problem-solving perspective, the paper structure follows fundamental stages for RES-based hybrid energy systems, including energy generation and demand, energy management, system analysis, optimisation, economic evaluation, and system performance^24^. These stages are realised through the proposed long-term modular modelling method (section "Proposed long-term modular modelling method"), economic modelling (section "Economic modelling"), the comprehensive set of metrics for the optimal sizing problem (section "Proposed comprehensive set of metrics for the optimal sizing problem"), the proposed sizing problem methodology (section "Proposed methodology for sizing problem"), and analysing techno-economic results for a case study (section "Case study and results analysis"). Finally, the section "Conclusion" concludes the paper.

## Proposed long-term modular modelling method

**Fig. 1:**
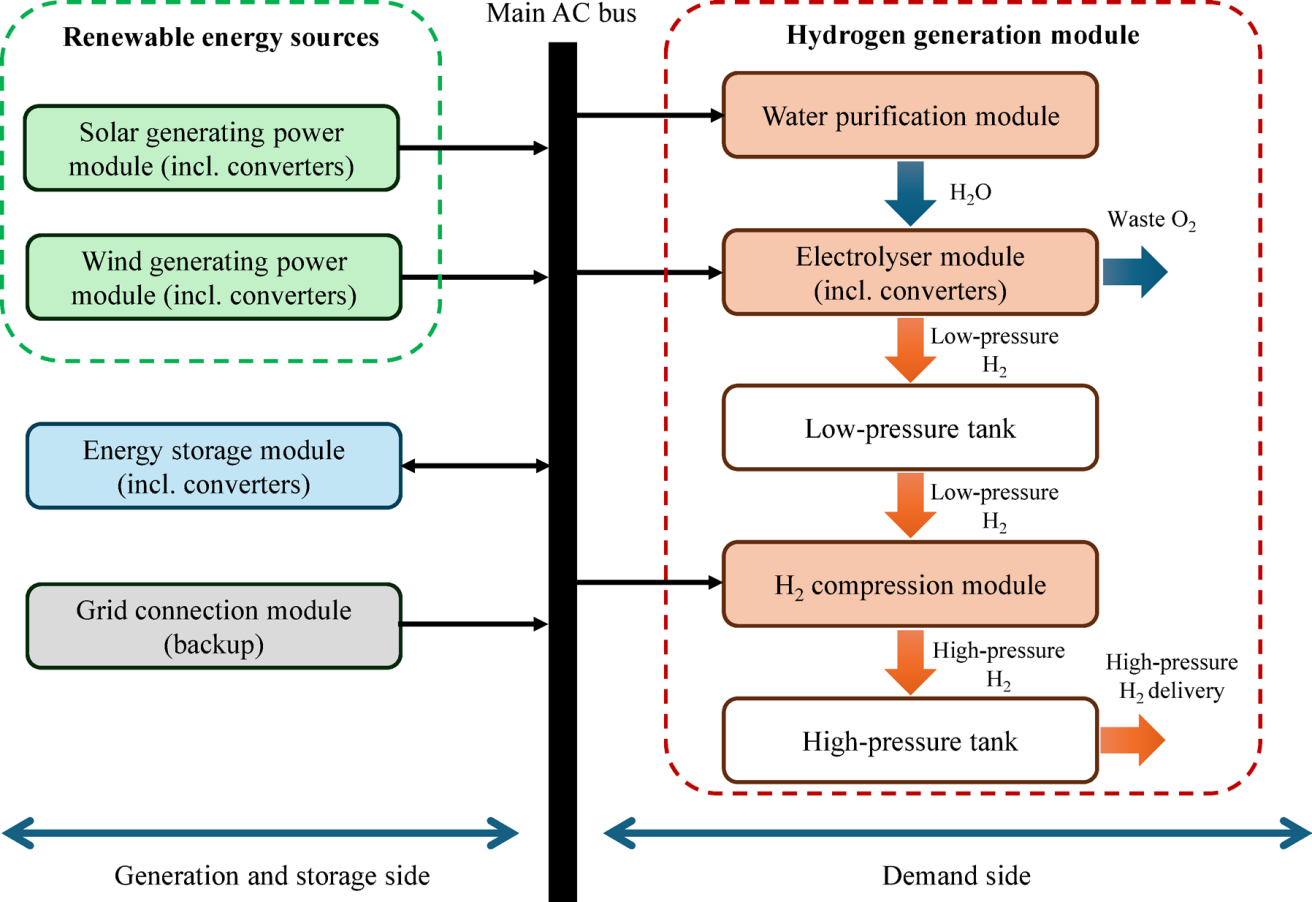
A schematic of the studied renewable power plant supplying a hydrogen generation system.

A schematic of the studied energy system is shown in Fig. 1. The generation and storage side includes solar and wind RESs, a grid connection as a backup energy resource, and a single energy storage system, which is assumed to be a lithium iron phosphate (LFP) battery. In most large-scale stationary energy storage system applications in recent years, the LFP battery technology is used because of several advantages, e.g., its high energy density, long cycle life, and decreasing production costs^25^.

The demand side is assumed to include the main energy demand terms of a hydrogen generation module (HGM), including the electrolyser module, the compressor module, and the water purification module. The main consumer of energy in an HGM is the electrolyser module, including one or more electrolysers, forming around 70–80% of the total energy demand. The hydrogen compression module may also include one or more compressors with different outlet pressures, which form 10–20% of the total HGM energy consumption. The main energy consumption of the water purification module belongs to the water pump(s), which can be considered as the third main consumer of energy. All converter and transformer energy consumptions are their losses, which are considered as a part of the corresponding module in the studied system, e.g., the consumption of the required converters and transformers for the electrolyser module is assumed as a part of these module losses.

Since sizing the renewable power plant modules of the HGM needs energy and power metrics modelling from the technical viewpoint, the detailed electrical modelling to model voltage, frequency, and current metrics, including very fast, fast, and medium dynamics, is not required. Instead, long-term modelling is performed for each module of the studied system, where the main modelled signal is the electrical power in an hourly resolution. Any conversion to/from the electrical power is a part of the modelling shown in detail in the next subsections. Such a long-term modelling method can be used for any suitable period of time, according to the study requirements, whether medium periods like one year or very long periods, such as the project lifetime.

Although some of the modules of the studied system have been modelled in literature and the presented models do not include considerable novelty, the unified modelling framework covering all modules is useful to facilitate using the proposed modelling method for a wide range of case studies. The models of wind and solar power generation modules are straightforward without any remarkable novelty. The model of the energy storage module, developed by the authors in another paper, is used here with a brief explanation of its operation. The grid connection module model is developed for the first time in this paper. Although there is a reasonable similarity in some parts of the set of models for the HGM with the existing models based on the nature of modules, they are basically original models developed in this paper, especially in a unified presentation form.

### Wind power generation module

In feasibility studies, there are two data-based modelling methods of the wind power generation modules, including the power-wind speed relationship and power curve methods, which are both based on wind speed data. The first one needs the power coefficient of the wind turbine, which is generally not provided by wind turbine manufacturers. Moreover, the wind turbine/generator speed and power limitations need to be considered, while all these limitations are considered in the power curve of the wind turbine. In addition, the power curves of most wind turbines in the market are validated through laboratory tests and power data of pre-installed samples. Therefore, the power curve modelling method is used here.

**Fig. 2:**
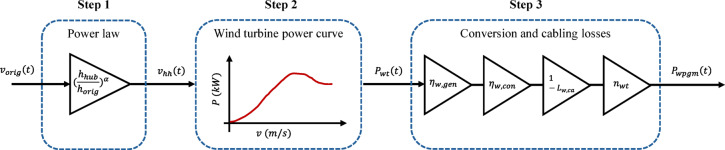
The modelling process of the wind power generation module.

Figure 2 shows the modelling process of the wind power generation module through three main steps. The wind speed data is usually measured or estimated in satellite-based data at 10 m height, which is not necessarily the same as the hub height of the wind turbine. Therefore, a conversion using one of the power or logarithmic laws is required to calculate the hub height wind speed. In the first step, the hub height wind speed ($\:{v}_{hh}$) is obtained from the original wind speed data ($\:{v}_{orig}$) using the power law as follows:

1 $$\:{v}_{hh}\left(t\right)={\left(\frac{{h}_{hub}}{{h}_{orig}}\right)}^{\alpha\:}\times\:{v}_{orig}\left(t\right),$$

where $\:\left(t\right)$ shows the wind value are a function of time, $\:{h}_{hub}$ is the hub height, $\:{h}_{orig}$ is the height at which the original wind speed data is measured/estimated, and $\:\alpha\:$ is the wind shear exponent to consider the terrain type.

In the second step, the power curve of the wind turbine is used to calculate the wind power of the wind turbine ($\:{P}_{wt}$) as follows:

2 $$\:{P}_{wt}\left(t\right)={f}_{pc}\left({v}_{hh}\left(t\right)\right),$$

where $\:{f}_{pc}$ is a nonlinear function to model the power curve, which is usually generated from the power curve provided by the wind turbine manufacturer using an interpolation method. Finally, in the third step, the output power of the wind power generation module ($\:{P}_{wpgm}$) is calculated from the wind turbine power as below:

3 $$\:{P}_{wpgm}\left(t\right)={n}_{wt}\times\:(1-{L}_{w,ca})\times\:{\eta\:}_{w,con}\times\:{\eta\:}_{w,gen}\times\:{P}_{wt}\left(t\right),$$

where $\:{n}_{wt}$ is the number of wind turbines in the wind power generation module, $\:{L}_{w,ca}$, $\:{\eta\:}_{w,con}$, and $\:{\eta\:}_{w,gen}$ are the cabling losses, converter efficiency, and generator efficiency in %/100 for each wind turbine, respectively.

### Solar power generation module

To calculate the power profile of the solar power generation module, solar irradiance terms on the inclined panels are required including diffuse ($\:{I}_{dif}$), direct ($\:{I}_{dir}$), and reflect ($\:{I}_{ref}$) irradiances. Although finding these irradiances on inclined surfaces can be challenging and is the topic of some literature, some satellite-based databases like PVGIS^26^ can estimate them, which can be used easily to find the solar power ($\:{P}_{spgm}$) as follows:

4 $$\:{P}_{spgm}\left(t\right)={n}_{pan}\times\:(1-{L}_{s,ca})\times\:{\eta\:}_{s,con}\times\:{\eta\:}_{pan}\times\:{S}_{pan}\left(t\right)\times\:({I}_{dif}\left(t\right)+{I}_{dir}\left(t\right)+{I}_{ref}\left(t\right)),\:$$

where $\:{n}_{pan}$ is the total number of panels, $\:{L}_{s,ca}$, is the total losses of cabling, wiring and dirty panels in %/100, $\:{\eta\:}_{s,con}$, and $\:{\eta\:}_{pan}$ are the converter and panel efficiency of the solar power generation module in %/100, and $\:{S}_{pan}\left(t\right)$ is the panel area in m^2^. All irradiance terms on the inclined panel are in kW/m^2^ and should be obtained considering the location latitude and longitude, the panel tilt angle, i.e., the angle of the panel from the horizontal plane, and the azimuth, i.e., the angle of the panel relative to the direction due South. In the case of having several parts of the solar farm with different tilt and azimuth angles, the solar irradiances should be obtained separately and then used in (4).

### Energy storage module

**Fig. 3:**
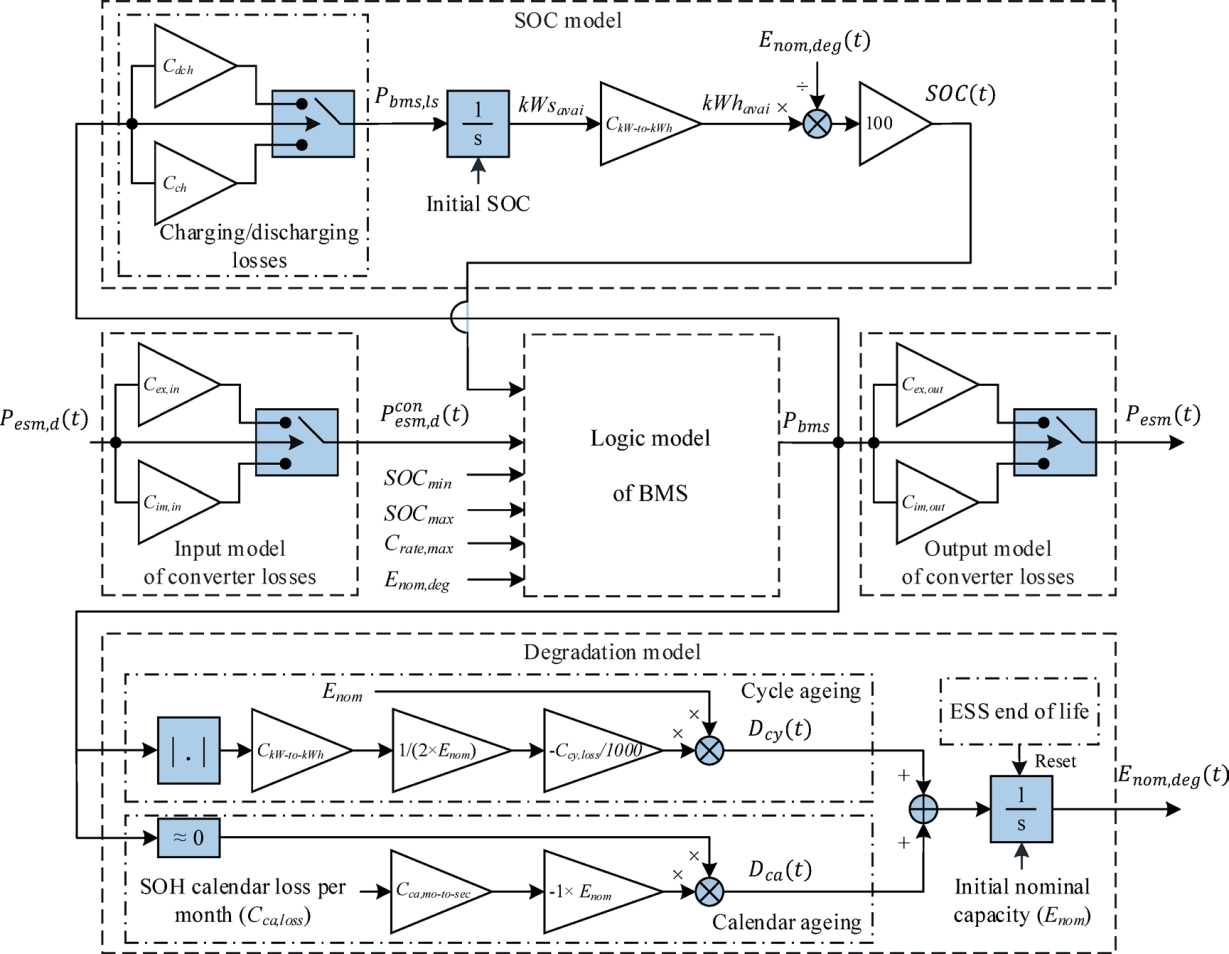
A schematic of the energy storage module modelling including all effecting parts on long-term performance.

Figure 3 shows a comprehensive schematic of the energy storage module (ESM) modelling, where the main input to the model is the charging/discharging power demand ($\:{P}_{esm,d}$) and the output is the actual power of the ESM ($\:{P}_{esm}$) considering all limitations affecting long-term performance, e.g., low and high limits of the state of charge (SOC). Although the model is a general parametric long-term model useful for different types of electrical and chemical energy storage systems for which the parameters should be obtained according to the manufacturers’ datasheets, it is most simplified to model the LFP battery storage systems. The ESM includes a bidirectional converter unit, LFP modules, and a battery management system (BMS). Accordingly, five main parts, namely the input model of converter losses, the local BMS model, the SOC model, the degradation model, and the output model of converter losses, are considered for the ESM model to model the most important long-term behaviours of the ESM.

The ESM power demand is a combination of charging and discharging power demands, where negative (positive) values are contracted to show the charging (discharging) power demand. Similarly, the actual ESM power and all internal power signals have the same logic, i.e., negative (positive) values show the actual charging (discharging) power. Therefore, the arrows in Fig. 3 do not show the direction of the charging/discharging power, but they show the flow of unreal signals modelling charging/discharging power.

The $\:{P}_{esm,d}$ is affected by the converter efficiency to obtain the net ESM demand ($\:{P}_{esm,d}^{con}$). The limitations that are checked in the BMS logic model include the low and high SOC limits, $\:{SOC}_{min}$ and $\:{SOC}_{max}$, and the maximum c-rate ($\:{C}_{rate,max}$). Only the net charging (discharging) power demand is accepted to effect on the SOC, if it does not cause a SOC greater (lower) than $\:{SOC}_{max}$ ($\:{SOC}_{min}$). Moreover, only a part of the net charging (discharging) power demand is accepted that is less than or equal to the maximum allowable charging (discharging) power, which is a multiplication of the degraded ESM capacity ($\:{E}_{nom,deg}$) and the $\:{C}_{rate,max}$. The BMS module output power ($\:{P}_{bms}$), considering all the ESM limitations, passes through the charging/discharging losses module to be decreased (increased) according to the battery charging (discharging) efficiency to obtain the battery internal charging (discharging) power, i.e., $\:{P}_{bms,ls}$. In the SOC model, the ESM SOC can easily be modelled by feeding an integrator by $\:{P}_{bms,ls}$ and considering the $\:{E}_{nom,deg}$. Note that the $\:{E}_{nom,deg}$ is modelled itself in the degradation model from the initial nominal capacity ($\:{E}_{nom}$) considering linear models of cycle and calendar ageing processes using their coefficients, which can be found in the manufacturer’s ESM datasheets. An integrator is required to find the $\:{E}_{nom,deg}$, where its initial value is set by the $\:{E}_{nom}$ and the sum of cycle and calendar ageing terms ($\:{D}_{cy}\:and\:{D}_{ca}$) is applied on its input. The integrator can be reset using its end of life to start a new period again with the $\:{E}_{nom}$. The details of the ESM modelling are available in^27^.

### Grid connection module

The energy provided through the grid connection can be generated by traditional fossil fuel power plants, the grid-connected large-scale RESs, e.g., solar farms and offshore wind farms, or a combination of both. This can be managed through energy/power purchase agreements between the RES power plant owner and the local supplier^28,29^. In any of these cases, the technical model of the grid connection is the same as presented in this section. However, the tariffs used in the economic model are different for each case.

A grid connection can be modelled considering its demand, constraints, and a control unit to determine the grid operation. Here, the grid connection module (GCM) is assumed to be operated in two situations: (1) to be used as a backup when the available RES power ($\:{P}_{res,av}$) is not enough to provide the HGM demand power ($\:{P}_{hgm,d}$), and (2) to support the ESM to raise its SOC from $\:SO{C}_{\mathrm{m}\mathrm{i}\mathrm{n}}$ for later use to meet the HGM demand. The total grid connection module power demand ($\:{P}_{gcm,d}$), is limited by its rated power ($\:{P}_{gcm,r}$). The losses of the substation can be modelled; however, it is less than 1% according to IEC 60076-20 for medium and big distribution transformer. Therefore, it is neglected in the model.

**Fig. 4:**
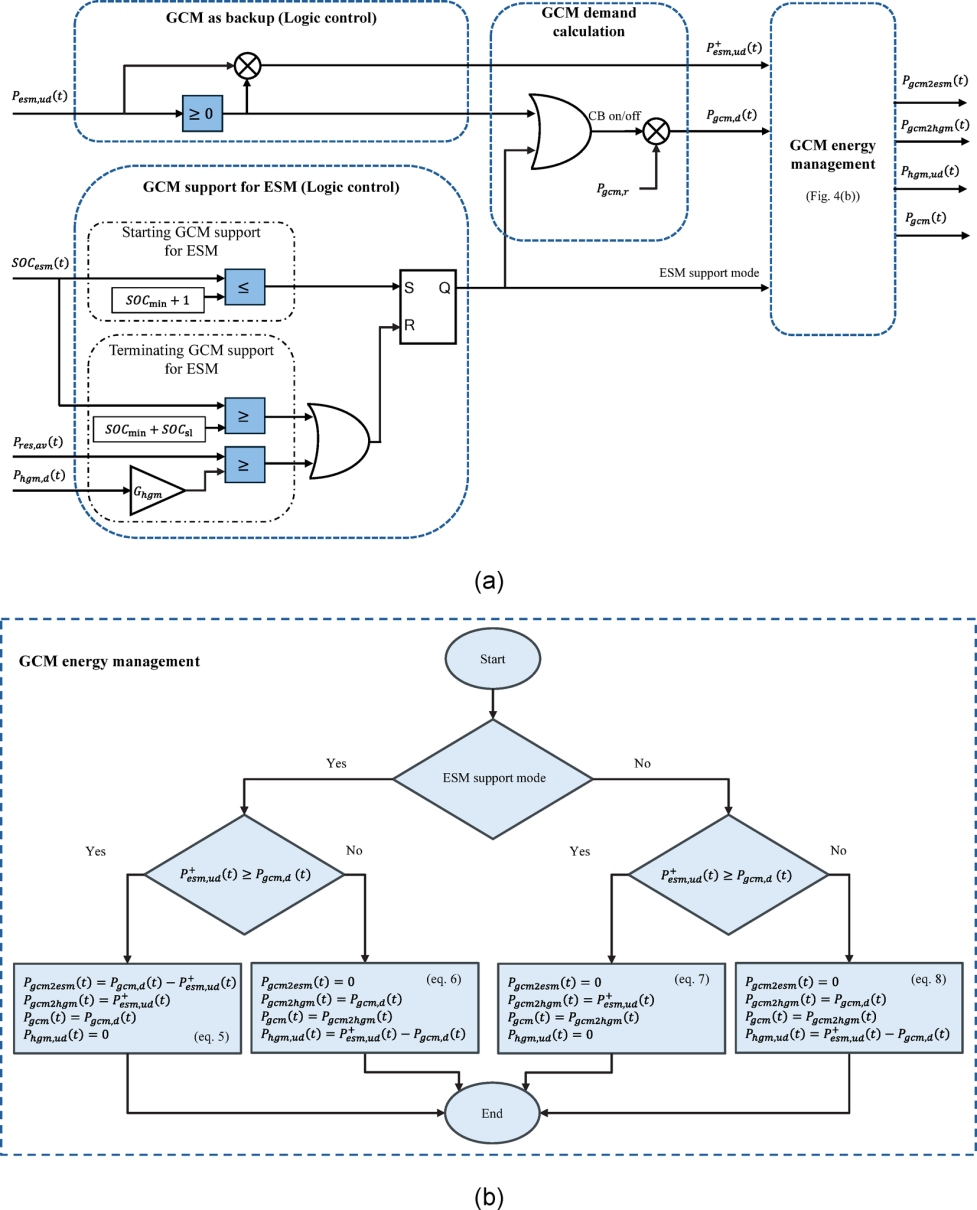
Grid connection module modelling, including: **a** logic control and interconnection between sub-modules and **b** grid energy management flowchart.

Figure 4(a) shows the sub-modules and their interconnection in the model of the grid connection module. The logic control to use the GCM as a backup is fed by the ESM unmet power demand to find its positive values ($\:{P}_{esm,ud}^{+}$), i.e., a part of the power demand which is not met neither by RESs nor by the ESM, as well as the corresponding time periods, which is a logic signal for switching on the grid connection circuit breaker. Another signal from the logic control for supporting the ESM can switch on the circuit breaker and operate the GCM with a demand equivalent to the rated power, i.e., $\:{P}_{gcm,d}\left(t\right)$=$\:{P}_{gcm,r}$. The ESM support by GCM starts when $\:SO{C}_{\mathrm{e}\mathrm{s}\mathrm{m}}\le\:SO{C}_{\mathrm{m}\mathrm{i}\mathrm{n}}+1$ and it is terminated if $\:SO{C}_{\mathrm{e}\mathrm{s}\mathrm{m}}$ increases to a predetermined support level ($\:SO{C}_{\mathrm{s}\mathrm{l}}$), or when the available RES power ($\:{P}_{res,av}$) is greater than the HGM demand power ($\:{P}_{hgm,d}$) assuming a margin gain ($\:{G}_{hgm}$) for the demand. A SR flip-flop is used to integrate all digital commands in the logic control of the ESM support sub-module and its output is a logic signal to show the status of the ESM support by the GCM and called ESM support mode. When both logic signals from both logic controls are zero, the circuit breaker switches off and $\:{P}_{gcm,d}\left(t\right)$=$\:0$.

The GCM energy management sub-module is the most important part of the GCM model, where receives $\:{P}_{esm,ud}^{+}$, $\:{P}_{gcm,d}$, and the ESM support mode to reasonably determine the direct GCM power to the HGM ($\:{P}_{gcm2hgm}$) and the GCM power to ESM ($\:{P}_{gcm2esm}$). Therefore, the total actual power provided by the grid is the sum of these two powers, i.e., $\:{P}_{gcm}={P}_{gcm2hgm}+{P}_{gcm2hgm}$, and the unmet HGM demand can be calculated separately for each situation according to the sub-module inputs. Figure 4(b) shows a flowchart including the detailed logic relationships and power calculations of the GCM energy management sub-module in (5)-(8).

### Hydrogen generation module

Although the main modules of the HGM related to electrical power modelling were introduced earlier including the electrolyser, hydrogen compression, and water purification modules, the HGM needs a low-pressure tank (LPT) buffering hydrogen for flexible operation of electrolyser and compressor modules and a high-pressure tank (HPT) to store hydrogen. Since, all these modules in an HGM are working together through electrical/control/hydraulic interconnections to generate, store, and deliver hydrogen, they should be modelled to find the total HGM power demand ($\:{P}_{hgm,d}$) from the corresponding hydrogen mass rate demand ($\:{\dot{m}}_{hgm,d}$).

#### Electrolyser and water purification modules

**Fig. 5:**
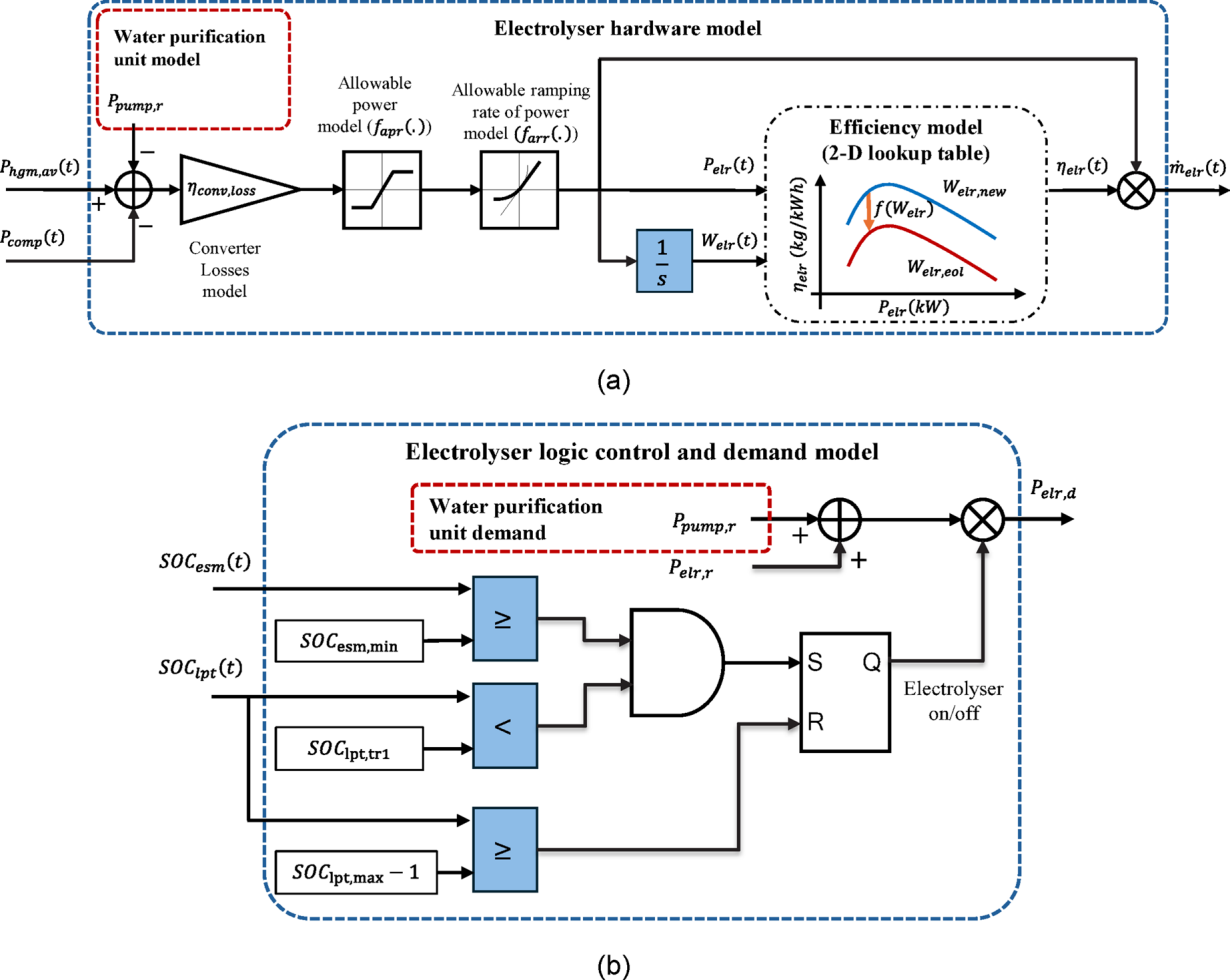
Electrolyser modelling: **a** hardware modelling, and **b** logic control and demand modelling.

Since the water purification module model is diminished to its pump electrical power modelling, it is modelled with the electrolyser module. Figure 5(a) shows the electrolyser hardware modelling. The main output of the electrolyser module is its generated hydrogen, which is modelled as a mass rate ($\:{\dot{m}}_{elr}$) and can be calculated as follows:

9 $$\:{\dot{m}}_{elr}\left(\mathrm{t}\right)={\eta\:}_{elr}\left(t\right)\times\:{P}_{elr}\left(t\right),$$

where $\:{\eta\:}_{elr}$ is the electrolyser efficiency in kgH_2_/kWh and $\:{P}_{elr}$ is the electrolyser internal power, which is used to electrolyze water. The electrolyser efficiency is affected by its operating point and the state of health (residual life) of the electrolyser stack. The $\:{\eta\:}_{elr}$-$\:{P}_{elr}$ relationship is usually provided through the corresponding curve by the manufacturer. Therefore, using a lookup table to model the $\:{\eta\:}_{elr}$-$\:{P}_{elr}$ curve and by importing the $\:{P}_{elr}\left(t\right)$, the efficiency can be calculated.

On the other hand, the end of life (EOL) of the electrolyser stack and the efficiency estimated values at the EOL can also be found in manufacturer datasheets and manuals. The $\:{\eta\:}_{elr}$-$\:{P}_{elr}$ curve can be easily calculated at the beginning and end of stack life shown by $\:{W}_{elr,new}$ and $\:{W}_{elr,eol}$ in Fig. 5(a). The accumulative energy usage of the electrolyser ($\:{W}_{elr}$) is a signal used to show the left life of the stack. Considering another lookup table to model $\:{\eta\:}_{elr}$-$\:{W}_{elr}$ relationship and by importing $\:{W}_{elr}\left(t\right)$, the efficiency can be calculated. Therefore, a 2-D lookup table is required to consider both $\:{\eta\:}_{elr}$-$\:{P}_{elr}$ and $\:{\eta\:}_{elr}$-$\:{W}_{elr}$ relationships at the same time and calculate the efficiency affected by both the operating point and the stack left life.

The electrolyser internal power needs to be calculated considering the conversion losses ($\:{\eta\:}_{conv,loss}$), the allowable ramping rates and the allowable power bands of the electrolyser as follows:

10 $$\:{P}_{elr}\left(t\right)={f}_{arr}\left({f}_{apb}\left({\eta\:}_{conv,loss}\times\:\left({P}_{hgm,av}\left(t\right)-{P}_{comp}\left(t\right)-{P}_{pump}\left(t\right)\right)\right)\right),$$

where $\:{f}_{arr}\left(.\right)$ is a normal rate limiter to model the allowable ramping rates and $\:{f}_{apb}\left(.\right)$ is a normal saturation limiter to model the allowable power bands of the electrolyser input power. The lower power band is to guarantee a minimum current flow level due to safety reason and the higher power band is the electrolyser rated power ($\:{P}_{elr,r}$). The maximum/minimum ramping rate is calculated according to the fastest power decrease/increase, which all can be found in the manufacturer datasheets. The $\:{P}_{hgm,av}$ is the total available power for the HGM, the $\:{P}_{comp}$ is the compressor actual power and both are obtained from other modules. The $\:{P}_{pump}$ is the pump actual power, which is assumed the same as the pump rated power ($\:{P}_{pump,r}$).

The electrolyser needs a logic control to determine the electrolyser demand ($\:{P}_{elr,d}$) according to the ESM and LPT SOCs. Figure 5(b) shows the logic control and demand modelling block. The electrolyser and water purification units switch on when there is enough electrical energy checked by the ESM SOC, i.e., $\:{SOC}_{esm}\ge\:{SOC}_{esm,min}$ and the LPT SOC has been decreased to a certain threshold known as $\:{SOC}_{lpt,tr1}$, i.e., $\:{SOC}_{lpt}<{SOC}_{lpt,tr1}$. Their operation terminates when the LPT is full modelled as $\:{SOC}_{lpt}\ge\:{SOC}_{lpt,tr1}-1$. The electrolyser demand is obtained by multiplying the electrolyser on/off logic signal by the total of the $\:{P}_{elr,r}\:and\:{P}_{pump,r}$.

#### Low/high pressure tank modules

**Fig. 6:**
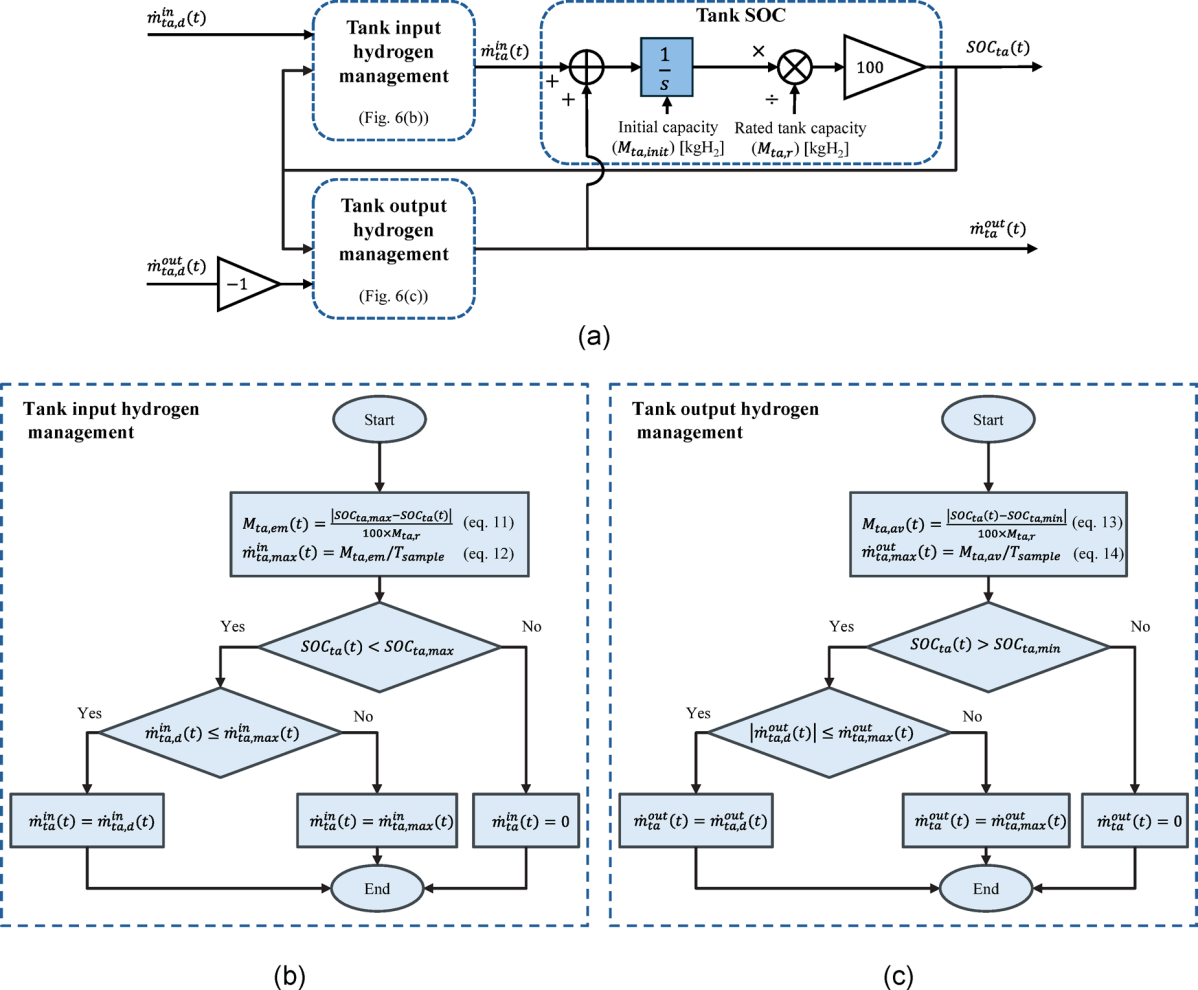
Schematic of the hydrogen tank model: **a** interconnection between the tank input/output hydrogen management and the tank SOC sub-modules, **b** the tank input hydrogen management model, and **c** the tank output hydrogen management model.

Since both LPT and HPT have the same governing relationships to be modelled in a long-term model, this subsection presents a unique model for them. Each hydrogen tank model includes three sub-modules consisting of tank input and output hydrogen management sub-modules and a tank SOC model as shown in Fig. 6(a). The tank input hydrogen management sub-module receives an input mass rate demand ($\:{\dot{m}}_{ta,d}^{in}$) and according to the SOC situation shown in Fig. 6(b) determines the actual input mass rate of the tank ($\:{\dot{m}}_{ta}^{in}$). The empty tank capacity ($\:{M}_{ta,em}\left(t\right)$) is calculated in (11) each sample time and accordingly the maximum allowable input mass rate ($\:{\dot{m}}_{ta,max}^{in}$) is calculated in (12). The $\:{\dot{m}}_{ta}^{in}$ can accept the total demand, i.e., $\:{\dot{m}}_{ta,d}^{in}$, only when $\:{SOC}_{ta}<{SOC}_{ta,max}$ and $\:{\dot{m}}_{ta,d}^{in}\le\:{\dot{m}}_{ta,max}^{in}$.

The tank output hydrogen management sub-module receives an output mass rate demand ($\:{\dot{m}}_{ta,d}^{out}$) and according to the SOC situation shown in Fig. 6(c) determines the actual output mass rate of the tank ($\:{\dot{m}}_{ta}^{out}$). The available tank capacity ($\:{M}_{ta,av}\left(t\right)$) is calculated in (13) each sample time and accordingly the maximum allowable output mass rate ($\:{\dot{m}}_{ta,max}^{out}$) is calculated in (14). The $\:{\dot{m}}_{ta}^{out}$ can accept the total demand, i.e., $\:{\dot{m}}_{ta,d}^{out}$, only when $\:{SOC}_{ta}>{SOC}_{ta,min}$ and $\:{\dot{m}}_{ta,d}^{out}\le\:{\dot{m}}_{ta,max}^{out}$. Other two situations can be seen in the flowchart in Fig. 6(c).

Finally, the tank SOC ($\:{SOC}_{ta}$) can be calculated as follows:

15 $$\:{SOC}_{ta}\left(t\right)=\frac{100}{{M}_{ta,r}}\left({\int\:}_{0}^{t}\left({\dot{m}}_{ta}^{in}\left(\tau\:\right)+{\dot{m}}_{ta}^{out}\left(\tau\:\right)\right)d\tau\:+{M}_{ta,init}\right),$$

where $\:{M}_{ta,r}$ is the rated tank capacity in kgH_2_ at the nominal pressure and $\:{M}_{ta,init}$ is the initial tank capacity, which can be calculated from the initial tank SOC ($\:{SOC}_{ta,init}$) as $\:{M}_{ta,init}={(SOC}_{ta,init}\times\:{M}_{ta,r})/100.$ Note that both the input/output hydrogen mass rates are considered with a plus in integrating, but the output hydrogen mass rate demand is multiplied by a −1 at the input of the corresponding management block.

#### Hydrogen compression module

**Fig. 7:**
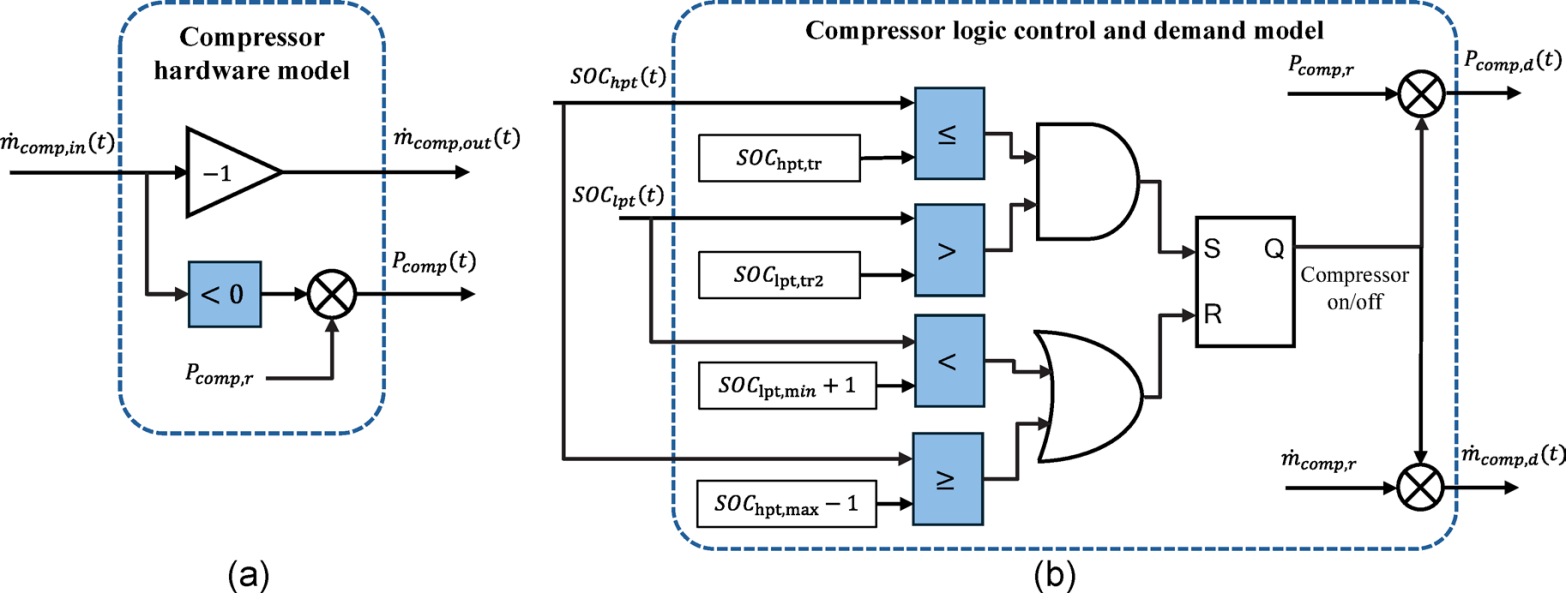
Compressor modelling: **a** hardware modelling, and **b** logic control and demand modelling.

There are different types of compressors including V, Z, D, and L types, which are different in shape, pressure range, and flow rate. Each type can be fixed speed leading to a fixed hydrogen mass rate or variable speed for a more flexible operation, which needs an inverter drive. Assuming a fixed speed compressor, the compression hydrogen module can also be modelled using only manufacturer provided data including the rated power ($\:{P}_{comp,r}$), hydrogen volumetric flow rate, and inlet/outlet pressures. One can easily calculate the fixed hydrogen mass rate using the ideal gas law since the pressure, volume, and temperature of the inlet/outlet hydrogen are in the linear compression area for most of standard hydrogen compressors^30^. According to the law of conservation of mass, the inlet and outlet mass rates are the same and thus modelling a compressor hardware through its mass rate is straight forward. As shown in Fig. 7(a), the inlet and outlet compressor mass rates ($\:{\dot{m}}_{comp,in}\:and\:{\dot{m}}_{comp,out}$) are the same in magnitude. Since the compressor inlet mass rate comes from the LPT output and the compressor outlet mass rate goes to the HPT input, a minus sign is required to make these two variables compatible. In addition to the mass rate modelling, the compressor electrical power ($\:{P}_{comp}$) needs to be modelled, which according to being fixed speed, it is assumed to be the same as $\:{P}_{comp,r}$. The logic signal for finding the time periods the compressor works is obtained from the input mass rate, i.e., when there is an input mass rate, the compressor works.

A logic control is required to determine the compressor power demand ($\:{P}_{comp,d}$) and the corresponding mass rate demand ($\:{\dot{m}}_{comp,d}$). Figure 7(b) shows the compressor logic control and demand model. To switch on the compressor, both LPT and HPT should be in an appropriate situation, which can be detected using their SOC. A threshold is defined for the HPT SOC to determine the desired SOC from which the compressor starts to refill the HPT called $\:{SOC}_{hpt,tr}$. Another threshold needs to be defined for the LPT to assume a level higher than the minimum SOC to guarantee reliable and seamless operation of the compressor called $\:{SOC}_{lpt,tr2}$. Therefore, the compressor switches on when $\:{SOC}_{hpt}\left(t\right)\le\:{SOC}_{hpt,tr}$ and $\:{SOC}_{lpt}\left(t\right)>{SOC}_{lpt,tr2}$. In order to switch off the compressor, either $\:{SOC}_{hpt}\left(t\right)\ge\:{SOC}_{hpt,max}-1$ or $\:{SOC}_{lpt}\left(t\right)<{SOC}_{lpt,min}+1$.

#### Hydrogen system modules interconnection

**Fig. 8:**
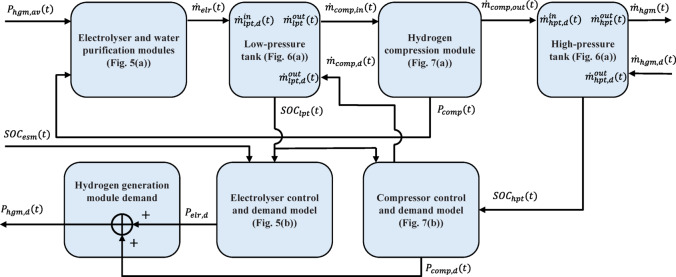
Module Interconnection of the hydrogen generation and storage system.

Figure 8 shows all required interconnections among the hydrogen generation and storage system modules, which have modelled earlier, to form the large module model at the demand side of the studied system known as the HGM model. All modules are referred to their corresponding models through the figures. In terms of the LPT and HPT modules, their internal variables are also shown to match the block with the general tank model shown in Fig. 6(a). Note that $\:lpt$ and $\:hpt$ are used instead of $\:ta$ in the subscript of the variables in Fig. 6(a) for LPT and HPT tank modules, respectively. From the electrical power modelling point of view, the HGM model has $\:{P}_{hgm,av}$ and $\:{SOC}_{esm}$ as inputs and $\:{P}_{hgm,d}$ as the main output. The hydrogen mass rate demand, i.e., $\:{\dot{m}}_{hgm,d}$, is the main input to the HGM as well as the overall studied system model, to determine hydrogen generation requirements. The actual produced hydrogen mass rate, i.e., $\:{\dot{m}}_{hgm}$, is the final output of the system, which will be used to calculate some important metrics.

### Modules interconnection

**Fig. 9:**
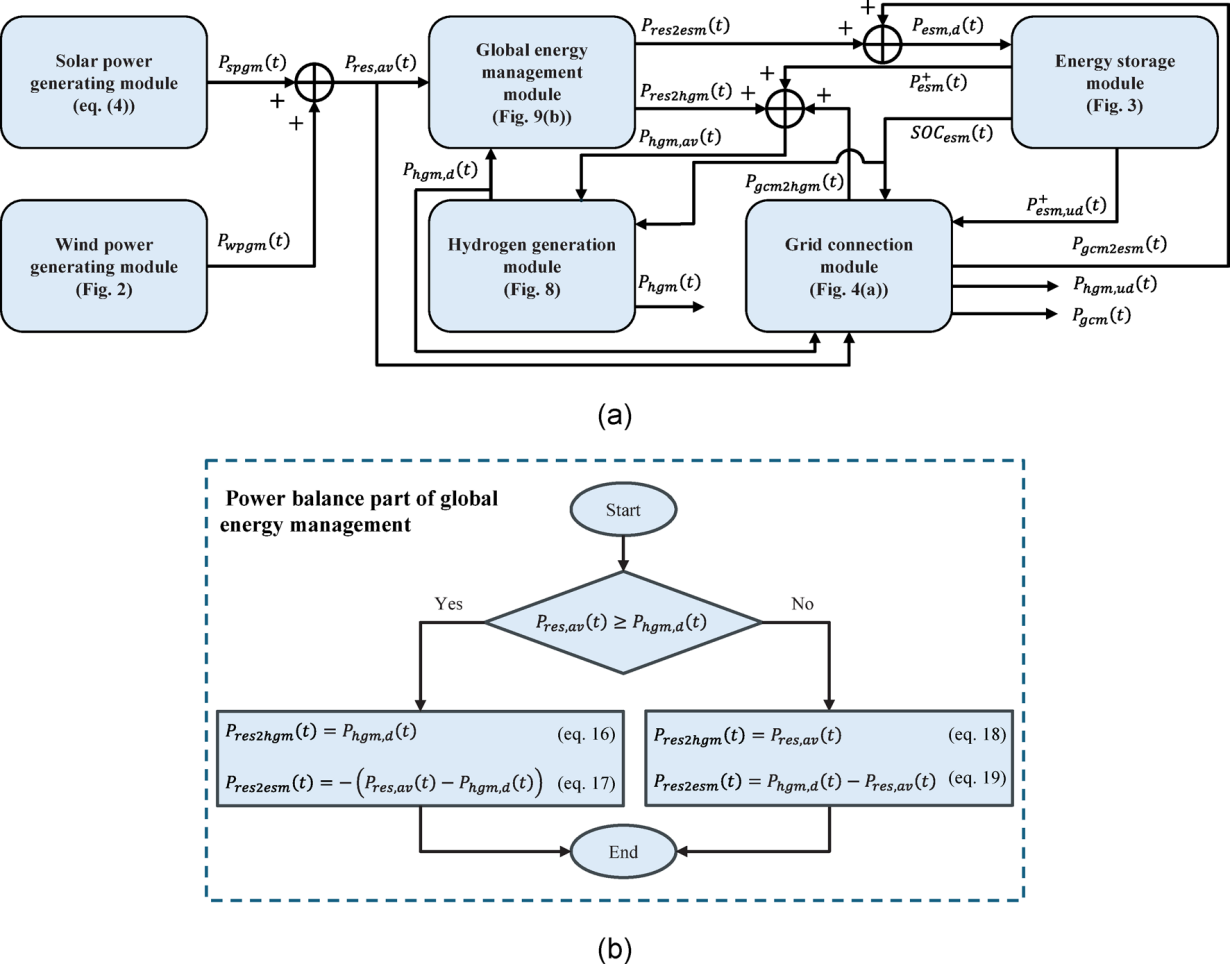
**a** Module interconnection of the studied renewable power plant supplying a hydrogen generation and storage system shown in Fig. 1, and **b** logic model of the power balance part of the global energy management module.

Figure 9(a) shows all interconnections among main modules of the studied system. Except the power balance part of the global energy management module, all other modules have earlier been modelled, and the corresponding figures and equations are referred inside the blocks. The global energy management model is shown in Fig. 9(b), where comparing the total available RES ($\:{P}_{res,av}$) and the total electrical power demand of the HGM ($\:{P}_{hgm,d}$), the direct RES power to the HGM ($\:{P}_{res2hgm}$) and the RES spillage for storing ($\:{P}_{res2esm}$) are calculated in (16)-(19). The ESM demand is the sum of $\:{P}_{res2esm}$ and $\:{P}_{gcm2esm}$. The total available power to supply the HGM ($\:{P}_{hgm,av}$) is the sum of direct RES power to the HGM ($\:{P}_{res2hgm}$), the direct GCM power to the HGM ($\:{P}_{gcm2hgm}$), and the discharged power from the ESM ($\:{P}_{esm}^{+}$).

From a control and operation viewpoint, the control system required for operating the studied system includes five separate parts that work together in a coordinated manner. They are the ESM’s local BMS (section "Energy storage module" and Fig. 3), the grid connection control unit (section "Grid connection module" and Fig. 4), the electrolyser control unit (section "Electrolyser and water purification modules.Electrolyser and water purification modules" and Fig. 5(b)), the compressor control unit (section "Hydrogen compression module.Hydrogen compression module" and Fig. 7(b)), and the global energy management between the RESs, ESM and the electrolyser (section "Modules interconnection" and Fig. 9(b)). Note that control coordination between the main modules of the studied system, which is necessary for reliable operation and is also another part of the global energy management, is modelled and explained in detail in the modelling of the control part of the modules, which are mentioned above in parentheses.

## Economic modelling

The economic modelling of the studied system is assumed to be through two metrics including CAPEX and LCOH. In CAPEX calculation, only the powerplant modules, i.e., the generation and storage side modules in Fig. 1, are considered because the demand side modules are assumed as existing assets. The total CAPEX in £ can be calculated as follows:

20 $$\:CAPEX\:\left(\pounds\:\right)=\:{\sum\:}_{k=1}^{{N}_{gssm}}{CAPEX}_{k},$$

where $\:{N}_{gssm}$ is the number of generation and storage side modules including solar and wind generation modules, the energy storage module and the backup grid connection. $\:{CAPEX}_{k}$ is the CAPEX of $\:k$’th module, which is generally obtained by multiplying its per-unit costs by the total installed capacity in unit, e.g., (£/kWp) $\:\times\:$ (kWp) for solar or wind power generation module.

To model LCOH, all modules of the studied system including hydrogen generation and storage system need to be considered. LCOH in £/kgH_2_ can be calculated as follows:

21 $$\:LCOH\:\left(\frac{\pounds\:}{kgH2}\right)=\:\frac{Present\:cost\:\left(\pounds\:/LT\right)}{{M}_{hgm,tot}\left(kgH2/LT\right)},$$

$\:LT$ is the project lifetime, $\:{M}_{hgm,tot}$ is the total produced hydrogen in the project lifetime, which can be obtained by integrating $\:{\dot{m}}_{hgm}\left(t\right)$. The $\:Present\:cost$ of the project is obtained as follows:

22 $$\:Present\:cost\:\left(\pounds\:/T\right)=\:{\sum\:}_{t=1}^{T}\frac{{C}_{t}}{{(1+i)}^{t-1}},$$

where $\:i$ is the discount rate, $\:{C}_{t}$ is the total of CAPEX and operation and maintenance expenses (OPEX) of all modules of the studied system in $\:t$’th year. To calculate the OPEX, the annual costs per unit should be multiplied by the total installed capacity in unit, e.g., (£/kWp/year) $\:\times\:$ (kWp/year) for solar or wind power generation module. Note that terms of annual costs per unit are different from one module to another, which will be presented in detail in the section "Case study and results analysis" and Table 5.

## Proposed comprehensive set of metrics for the optimal sizing problem

In this section, a set of technical, economic, and techno-economic metrics is introduced/proposed to be used by a renewable power plant designer to take into account different aspects of the design in the power plant sizing problem. In other words, the designer needs to consider different feasible combinations of sizes for the power plant modules, each one is called a plan and compare them through these metrics to find the best plan. To obtain power/energy-related variables for calculating technical and techno-economic metrics, the studied system is assumed to be simulated in one year to limit the simulation time. This duration seems to be enough to consider seasonal weather changes. Moreover, most of the other variables, like the hydrogen demand or annual production, can be easily calculated according to the one-year simulation, available data, or assumptions.

### Met hydrogen demand

The most important technical metric is the met hydrogen demand, which is used to find the percentage of total hydrogen demand that can be met by the power plant. It is calculated as:

23 $$\:MHD\:\left(\%\right)=\frac{Daily\:average\:hydrogen\:production\:(kgH2/day)}{Daily\:average\:hydrogen\:demand\:(kgH2/day)}\times\:100,$$

where the daily average hydrogen production/demand can be calculated by averaging the corresponding annual values. Although the stack degradation is modelled in the electrolyser module for one-year simulations, it should be modelled to consider its impact on the annual hydrogen production during the project lifetime. The annual hydrogen generation by the electrolyser for *j*’th year ($\:{M}_{H2,yj}$) is calculated through (24) from the one-year simulation ($\:{M}_{H2,y1}$), which is the first year of the stack use.

24 $$\:{M}_{H2,yj}(kgH2/y)=\frac{Max.\:daily\:H2\:production\:in\:{j}^{{\prime\:}}th\:year\:\left(\frac{kgH2}{day}\right)}{Max.\:daily\:H2\:production\:in\:first\:year\:\left(\frac{kgH2}{day}\right)}{M}_{H2,y1}(kgH2/y),$$

Note that the maximum daily hydrogen production in *j*’th year can easily be obtained using the maximum daily hydrogen production at the beginning and end of stack life from the datasheet and assuming a linear interpolation.

### Proposed RES energy use and installed power efficiencies

The RES energy use efficiency is proposed in (25) to evaluate how a plan can be efficient in terms of using the available renewable energy. A plan may have a very high MHD, but have a huge RES spillage, i.e., a low $\:{\eta\:}_{res,w}$.

25 $$\:{\eta\:}_{res,w}\:\left(\%\right)=(1-\frac{RES\:spillage\:energy\:\left(kWh\right)}{Total\:available\:RES\:energy\:\left(kWh\right)})\times\:100.$$

The RES spillage energy and total available RES energy can be calculated by integrating $\:{P}_{esm,ud}^{-}\left(t\right)$, and $\:{P}_{res,av}\left(t\right)$, respectively.

Regarding RES, another efficiency can be proposed to evaluate the plan suitability considering the RES installed power instead of RES energy usage as follows:

26 $$\:{\eta\:}_{res,p}\:\left(\frac{kgH2}{year}/kWp\right)=\frac{Annual\:H2\:generation\:(kgH2/year)}{Total\:installed\:renewable\:power\:\left(kWp\right)},$$

where the total installed renewable power is the sum of installed peak power of the wind and solar generating power modules, and the annual hydrogen generation is easily calculated by averaging $\:{M}_{H2,yj}$, $\:j=1,\:\dots\:,\:LT$.

### Proposed ESM installed capacity efficiency and SOH

Like (26), (27) is trying to evaluate the efficiency of using the installed ESM capacity.

27 $$\:{\eta\:}_{esm}\:\left(\frac{kgH2}{year}/kWh-ESM\right)=\frac{Annual\:H2\:generation\:(kgH2/year)}{ESM\:installed\:capacity\:(kWh-ESM)}.$$

Moreover, the ESM state of health (SOH) is important to be considered to support plans that lead to a higher SOH. The ESM SOH is calculated as follows:

28 $$\:{SOH}_{esm}\:\left(\%\right)=\frac{Degraded\:ESM\:capacity\:\left(kWh\right)}{Nominal\:ESM\:capacity\:\left(kWh\right)}\times\:100,$$

where the degraded ESM capacity is calculated using the ESM model in the section "Energy storage module". Since the simulation period is one year and both the cycle and calendar ageing processes are linear, one can calculate the degraded ESM capacity for any period of operation using the one-year degraded capacity. Another point that needs to be considered in calculating the degraded ESM capacity is the type of actions against capacity degradation, e.g., replacement, augmentation^31^, and reconfiguration^32,33^. Here, an augmentation is assumed to be done every ten years to raise the degraded capacity to its nominal/initial capacity. Therefore, the degraded ESM capacity should be calculated within a ten-year period.

### Grid share of MHD

To generate more green hydrogen and to limit greenhouse gas emissions, the grid connection share of MHD (GCS) is interested to be evaluated by (29), where the grid used energy, and the RES used energy are calculated by integrating $\:{P}_{gcm}\left(t\right)$, and $\:{P}_{hgm}\left(t\right)$, respectively.

29 $$\:GCS\:\left(\%\right)=\frac{Grid\:used\:energy\:\left(\frac{kWh}{year}\right)}{Total\:actual\:energy\:consumption\:\left(\frac{kWh}{year}\right)}\times\:100.$$

Note that GCS needs to be minimised to achieve a higher contribution from the local power plant. Furthermore, in the case of purchasing the grid energy from traditional non-RES power plants, GCS shows the percentage of the grey hydrogen from the total produced hydrogen.

### CAPEX and LCOH

CAPEX, as the only economic metric, and LCOH, as the only techno-economic metric, which are both very important in decision-making for finding the best plan, have already been defined in (20) and (21). Note that LCOH is calculated for the project lifetime. Since the technical overall model is performed for one year, the lifetime hydrogen generation in the LCOH formula, i.e., (21), is obtained by multiplying the hydrogen generated for the sample year by the number of years of the lifetime. On the other hand, all CAPEX and OPEX over the lifetime are considered in LCOH calculations.

## Proposed methodology for sizing problem

Usual methods to find the best plans for power plants supplying a hydrogen generation system employ CAPEX, LCOH or levelised cost of energy as a single-objective optimisation function, in addition to considering technical constraints like a minimum MHD. Therefore, the feasible/search space of the optimisation problem is set to the range of plans, which are compared together based on a single objective, after removing those plans that do not satisfy the constraints. The same process is employed here but considering a multi-objective cost function.

Pareto front optimisation approaches are a wide family of multi-objective optimisation methods that produce a set of trade-off solutions instead of only one final optimal solution^34,35^. The weighted sum method, as another multi-objective optimisation method, can support the idea of trade-off solutions by changing weighting coefficients, but with a simpler methodology and a lower calculation burden. In this paper, the weighted sum method is used, which works with changeable weighting coefficients. It is suitable for simple problems with a convex feasible space. Its simplicity and fast computation make it one of the most common multi-objective optimisation methods. But it cannot handle non-convex problems and requires a weighting coefficient tuning^36^. Multi-objective evolutionary algorithms are also another large group of Pareto front methods with their specific way to approach the set of solutions. In contrast, evolutionary multi-objective optimisation methods are much more powerful for large-scale engineering tasks with several optimisation variables, leading to complex and wide non-convex feasible spaces. They can explore the entire Pareto front, including complex shapes, in a single run, but at the cost of higher computation and algorithm complexity with respect to weighted sum methods.

For less-complicated optimisation problems with a limited search space, including a few optimisation variables, like optimal sizing problems with a small number of variables, the simplicity of implementation and operation of the weighted sum method is an advantage over more complicated evolutionary multi-objective optimisation methods. Although the weighted sum method needs tuning of weighting coefficients, this is not a big challenge for tangible engineering optimisation problems in which a set of reasonable coefficients can be found according to the nature of corresponding objectives^36^. Furthermore, sensitivity analysis on weighting coefficients can be employed to search for a wider range of solutions. The running time of the weighted sum methods is expected to be considerably lower than the running time of the evolutionary multi-objective optimisation methods for the same problem. Therefore, even rerunning the optimisation with different weighting coefficients does not make the total optimisation process too long compared to the evolutionary methods.

The proposed method is to use the set of metrics ($\:{M}_{norm}$) introduced/proposed in the previous section to form a multi-metric optimisation function ($\:{f}_{optimization}$) through a weighted sum as follows:

30 $$\:{f}_{optimization}\left({M}_{norm}\right)=\:{\sum\:}_{q=1}^{8}{c}_{m,q}\times\:{m}_{n,q},$$

where $\:{M}_{norm}=\left[{MHD}_{n}\:{\eta\:}_{res,w,n}\:{\eta\:}_{res,p,n}\:{\eta\:}_{esm,n}\:{SOH}_{esm,n}\:{GCS}_{n}\:{CAPEX}_{n}\:{LCOH}_{n}\right]$, and $\:n$ in subscript of each metric indicates a normalised value of that metric. Table 3 shows the steps required to normalise the metrics. All normalised metrics should have the same direction regarding the optimisation function, which is assumed to be maximised. Therefore, those metrics that needs to be maximised, required only Step 1 but others need all three steps. $\:nor(.)$ is the normalisation function to calculate a normalised metric ($\:{m}_{n,q}$) from its original metric ($\:{m}_{orig}$) as follows:

31 $$\:{m}_{n,q}=\:nor\left({m}_{orig,q}\right)=\frac{{m}_{orig,q}}{{m}_{orig,q}^{max}},$$

where $\:{m}_{orig,q}$ is the vector of original values of the $\:q$’th metric for all plans assumed in the search space and $\:{m}_{orig,q}^{max}$ is the maximum value of the vector. Therefore, $\:\mathrm{max}\left({m}_{n,q}\right)=1.$ The $\:{c}_{m,q}$ is the weighting coefficient for $\:q$’th normalised metric ($\:{m}_{n,q}$) such that $\:{\sum\:}_{q=1}^{8}{c}_{m,q}=1$. Therefore, $\:{f}_{optimization}$ is always less than or equal to 1. Plans with the largest value of $\:{f}_{optimization}$ can be selected as the optimised plans considering the abovementioned metrics. However, the weighting coefficients needs to be selected by the designer as an optimisation parameter. One can consider MHD, CAPEX, and LCOH as the high valuable metrics, $\:{\eta\:}_{res,w}$, and GCS as the medium valuable metrics, and other as the low valuable metrics in relation to each other. Methods like AHP can be used to calculate the weighting coefficients considering the determined importance level of metrics. Note that suitable constraints can also be considered in the optimisation problem, which will be discussed in the case study.

**Table 3:** Steps required for normalizing the metrics used in the optimisation function in (30).

Original metric	Optimisation direction	Step 1: first normalisation	Step 2: changing direction	Step 3: second normalisation
$\:MHD$	Maximization	$\:{MHD}_{n}=nor\left(MHD\right)$	-	-
$\:{\eta\:}_{res,w}$	Maximization	$\:{\eta\:}_{res,w,n}=nor\left({\eta\:}_{res,w}\right)$	-	-
$\:{\eta\:}_{res,p}$	Maximization	$\:{\eta\:}_{res,p,n}=nor\left({\eta\:}_{res,p}\right)$	-	-
$\:{\eta\:}_{esm}$	Maximization	$\:{\eta\:}_{esm,n}=nor\left({\eta\:}_{esm}\right)$	-	-
$\:{SOH}_{esm}$	Maximization	$\:{SOH}_{esm,n}=nor\left({SOH}_{esm}\right)$	-	-
$\:GCS$	Minimization	$\:{GCS}_{n1}=nor\left(GCS\right)$	$\:1-{GCS}_{n1}$	$\:{GCS}_{n}=nor(1-{GCS}_{n1})$
$\:CAPEX$	Minimization	$\:{CAPEX}_{n1}=nor\left(CAPEX\right)$	$\:{1-CAPEX}_{n1}$	$\:{CAPEX}_{n}=nor\left({1-CAPEX}_{n1}\right)$
$\:LCOH$	Minimization	$\:{LCOH}_{n1}=nor\left(LCOH\right)$	$\:1-{LCOH}_{n1}$	$\:{LCOH}_{n}=nor(1-{LCOH}_{n1})$

## Case study and results analysis

**Fig. 10:**
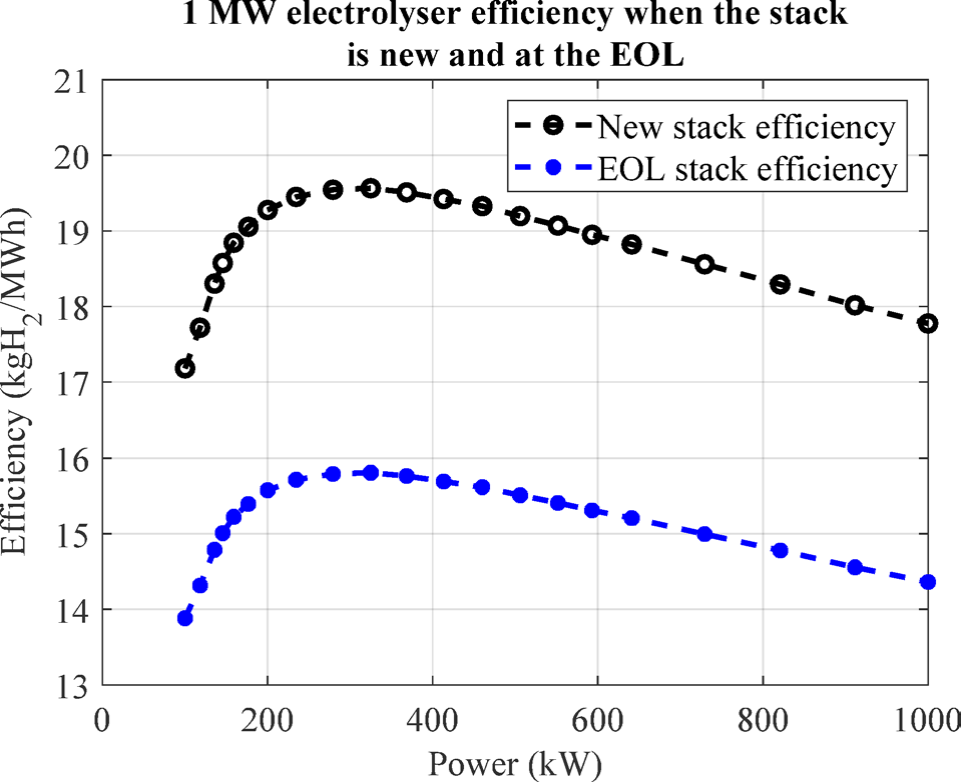
The 1 MW PEM electrolyser characteristic curve including efficiency when the stack is new and at its end of life (EOL)^37^.

Alongside providing the roadmap of the hydrogen generation, storage, and usage technologies for all related organisations and industries by policymakers in the UK, the demand for feasibility studies of potential cases is increasing. A stakeholder needs to know the renewable generation and storage requirements to operate a hydrogen generation and storage system, including a 1 MW electrolyser, as much as possible to maximise the hydrogen generation. The electrolyser is assumed to be an ITM electrolyser, and its technical data is available^37^ and can be seen in Table 4. The period of electrolyser stack replacement is 10 years. Figure 10 shows the electrolyser efficiency, which is taken from ITM datasheets^37^. A permanent hydrogen demand of 18 kgH_2_/h is assumed according to the maximum hydrogen generation operating point of the 1 MW electrolyser and the stakeholder requirement. The main modules required to achieve this goal are those shown in Fig. 1.

From the hydrogen system design point of view, compatible mass/flow rates and pressures should be considered to find a suitable compressor as well as a water pump to work with the 1 MW electrolyser, which is a 75 kW compressor^38^, and a 10 kW water pump. The low-pressure and high-pressure tanks are usually designed according to the long-term hydrogen demand. Since the hydrogen demand is permanent, and the demand side design is not the main goal of the paper, the tanks are assumed to be large enough for the full-time system operation in technical analysis. However, limited sizes are assumed in the LCOH calculations. See Tables 4 and 5 for more technical and economic data.

From the power plant design point of view, the land for solar and wind generation modules is assumed to be available for a capacity of three times the power demand, i.e., 3 MWp per technology. The location for gathering weather data is a rural area close to Sheffield, UK. The solar irradiances and wind speed data are obtained from the PVGIS database for 2023, which is an open-access satellite-based database^26^. The panel tilt angle and azimuth are assumed to be 25 degrees and 0 degrees, respectively. The panel model is a 595 Wp with 21.7% efficiency^39^. The height at which the wind speed data is estimated is 10 m/s. The wind turbine is ATB 500.54 from ATB Riva Calzoni with 500 kWp rated power. Required data is open access, including the power curve^40^. The ESM is an LFP battery. The period of ESM module augmentation is 10 years to provide the initial capacity again. Tables 4 and 5 show more information about the modules with the corresponding references.

The grid connection has two options of 200 kW and 500 kW as the rated power, which are provided by standard sizes of 315 kVA and 630 kVA substations. The grid connection charges include two fixed costs, the first per connection and the second per capacity, and two energy costs, the first per consumed kWh and the second per exceeded kVA. The costs per unit are available for all four costs^48^, but the consumed kWh per unit cost needs to be obtained as an average of three charges for red, yellow, and green time bands. Moreover, the exceeded kVA needs to be calculated using the formula available in^48^, and the power factor is assumed to be 0.96. These tariffs are for normal energy/power purchase agreements in which the supplier does not guarantee to provide the client’s energy from grid-connected RESs. Therefore, GCS shows the grey percentage.

**Table 4:** Technical details of the studied system modules.

Specifications	Value (unit)	Specifications	Value (unit)
**Hydrogen generation module**		**Solar power generation module** ^39^	
**Electrolyser** ^37^		Rated power	595 (Wp)
Rated power	1000 (10) (kW)	Panel efficiency	21.7 (%)
Water pump rated power	10 (kW)	Panel area	2.58 (m^2^)
Stack EOL	10 (y)	Converter efficiency	97 (%)
Cold start-up time	300 (s)	**Wind power generation module** ^40^	
Shut-down time	2 (s)	Rated power	500 (kWp)
Max. H2 production (EOL)	427 (kg/day)	Hub height	70 (m)
Max. H2 production (new)	345 (kg/day)	Generator/converter eff.	96/96 (%)
Output hydrogen pressure	10 (bar)	Cabling losses	2 (%)
**Compressor** ^38^		**Energy storage module**	
Rated power	75 (kW)	Converter import/export losses	3 (%)
Volumetric flow rate	250 (Nm^3^/h)	Charge/discharge losses	3 (%)
Inlet/suction pressure	10 (bar)	Maximum c-rate	1
Inlet H2 temperature	288 (K)	Initial SOC	50 (%)
Outlet pressure	300 (bar)	Minimum allowed SOC	5 (%)
**Low/High pressure tank**		Maximum allowed SOC	95 (%)
Minimum allowed SOC	5/1 (%)	Cycle ageing^41^	4.5 (%/1000 c)
Maximum allowed SOC	100 (%)	Calendar ageing^42^	0.125 (%/month)
Nominal pressure	10/300 (bar)	**Grid connection module**	
Rated capacity	40/100 (kg)	$\:{\:SOC}_{sl}$	35 (%)
$\:{SOC}_{hpt,tr},{\:SOC}_{lpt,tr1}$,$\:\:{SOC}_{lpt,tr2}$	20, 20, 50	$\:{\:G}_{hgm}$	1.2

**Table 5:** Terms of expenses and cost per unit used in CAPEX and OPEX calculations.

Term of expenses	Cost (unit)	Term of expenses	Cost (unit)
**Hydrogen generation module (HGM)** ^43^		Fixed O&M and insurance costs (OPEX)	7.1 (£/kW/y)
Electrolyser purchase costs (CAPEX)	1000 (£/kW)	**Energy Storage module**^45^^46^,	
Compressor costs (CAPEX)	150,000 (£/each)	LFP Battery and cabinet purchasing costs (CAPEX)	300 + 15 (£/kWh)
Low-pressure Tank costs (CAPEX)	750 (£/kg)	Inverter purchasing costs (CAPEX)	60 (£/kW)
High-pressure Tank costs (CAPEX)	1200 (£/kg)	Installation and BOS costs (CAPEX)	130 (£/kWh)
Electrical equipment costs (CAPEX)	100,000 (£)	Container 40 ft (CAPEX)	60,000 (£)
Balance of Plant costs (CAPEX)	0.15*HGSS CAPEX	BESS OPEX^45^	(3% of CAPEX)/y
HGM OPEX	70 (£/kW/y)	**Grid connection module**	
**Wind power generation module** ^44^		315 kVA substation (CAPEX)^47^	40,000 (£/each)
Pre-development costs (CAPEX)	130 (£/kW)	630 KVA substation (CAPEX)^47^	50,000 (£/each)
Construction costs (CAPEX)	1100 (£/kW)	Grid energy cost (OPEX)^48^	1.6 (£/MWh)
Fixed O&M and insurance costs (OPEX)	27.4 (£/kW/y)	315 kVA/630 kVA substation fixed charge^48^	3.61/8.66 (£/day)
**Solar power generation module** ^44^		315 kVA/630 kVA substation capacity charge^48^	0.086 (£/kVA/day)
Pre-development costs (CAPEX)	50 (£/kW)	315 kVA/630 kVA substation exceeded capacity charge^48^	0.086 (£/kVA/day)
Construction costs (CAPEX)	200 (£/kW)		

The integrated model of the studied system, with the configuration shown in Fig. 1, details of the control and operation modelled and elaborated in the section "Proposed long-term modular modelling method", and parameters and specifications presented above, is implemented in MATLAB. The system operation can be summarised as follows:

1. The constant 18 kgH2/h hydrogen demand is applied to the HGM, which leads to a decrease in the HPT hydrogen level. According to the compressor control rules (Fig. 7(b)), it starts to fill the HPT when the HPT SOC decreases below 20% and the LPT SOC is greater than 50%. The compressor stops if the HPT is full or if the LPT SOC reaches its minimum SOC, i.e., 5%.
2. According to the electrolyser control rules (Fig. 5(b)), when the LPT SOC decreases below 20% and the LFP battery SOC is greater than its minimum, i.e., 5%, the electrolyser starts to generate hydrogen directly from the RES power if it is available or from the LFP battery power. According to the power balance part of the global energy management (Fig. 9(b)), the battery covers RES power to allow the electrolyser to work at its greatest possible power unless its SOC reaches below the minimum value, i.e., 5%, controlled by its own BMS control (Fig. 3). If there is enough power from the renewable power plant to operate the electrolyser within its operating range, it does not stop unless the LPT is full.
3. According to the GCM energy management (the right branch of Fig. 4(b)), the grid connection backs up the RESs and LFP battery if they cannot meet the electrolyser rated power alone or together. Moreover, it starts supporting the LFP battery when there is no RES power, and the battery’s SOC reaches its minimum. It does not stop until the battery SOC reaches 35% or the RES power is greater than 1.2 times the HGM rated power (Fig. 4(a) and the left branch of Fig. 4(b)). According to the limited power of the grid connection, 200 kW or 500 kW, against the HGM rated power, e.g., 1085 kW when the electrolyser stack is new, HGM unmet demand is still probable, which will be considered in the metric calculations.

### Important profiles of the proposed modelling method

**Fig. 11:**
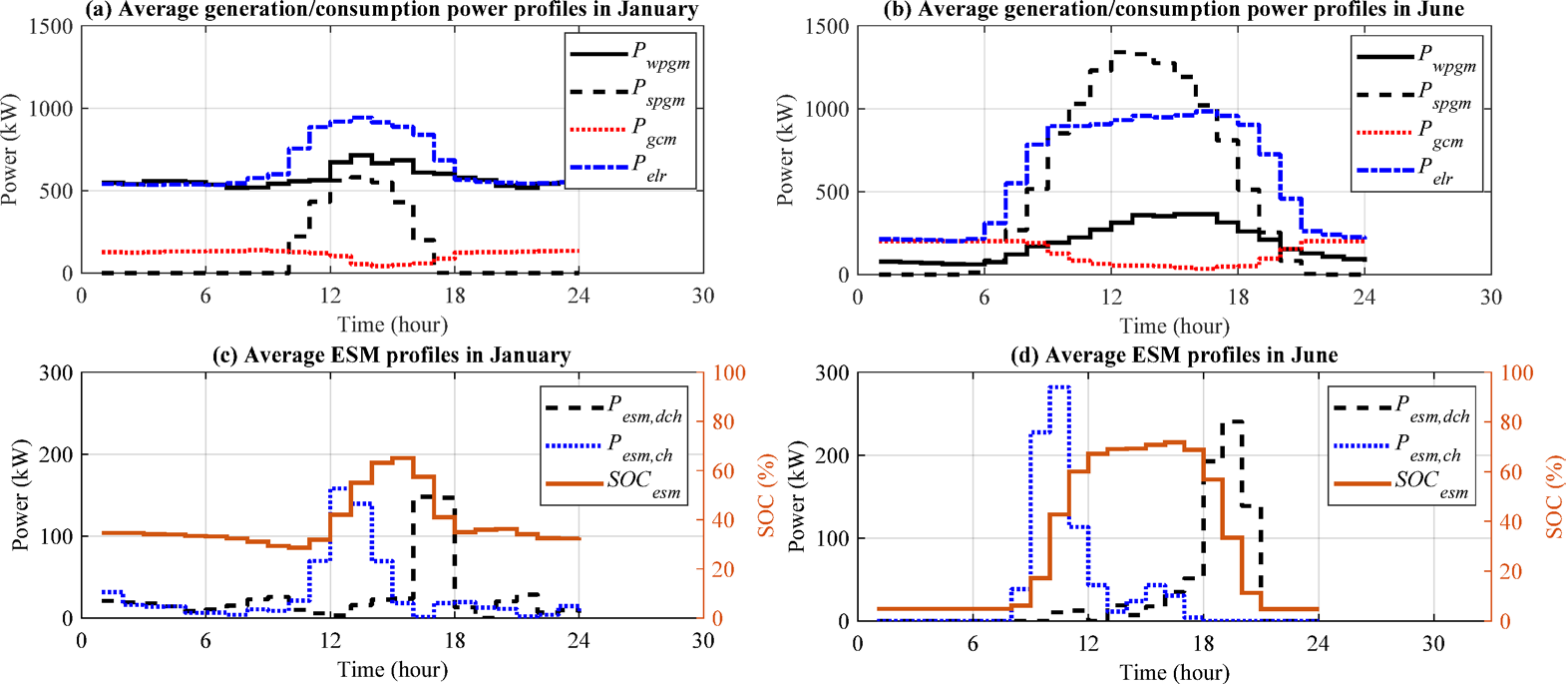
Important average profiles of the proposed modelling method: **a** RES (solid and dashed black), grid (dotted red) and consumption (dash-dotted blue) power profiles in January, **b** the same power profiles in June, **c** SOC (solid brown), and charging (dotted blue) and discharging (dashed black) power profiles of the ESM in January, and **d** the same profiles of the ESM in June.

Since the first-level outputs of the proposed modelling method are several powers, SOC, and hydrogen mass rate profiles for a long duration, showing all of them needs several pages, too long for a research paper. As a sample, a plan including 1.5 MWp installed wind power, 2.5 MWp installed solar power, 1 MWh ESM capacity and a 200 kW grid connection is simulated using the modelling method proposed in the section "Proposed long-term modular modelling method". and a summary of the most important profiles are shown in Fig. 11 including daily average power and SOC profiles for January and June, which are calculated by averaging all daily profiles of each variable in the corresponding months.

Even though the wind power profile ($\:{P}_{wpgm}$) is higher than the solar power profile ($\:{P}_{spgm}$) overall, 24 h in January (Fig. 11(a)), it is considerably lower than the solar power profile during the daytime in June (Fig. 11(b)). This allows the electrolyser to be operated at night in January with a high power consumption ($\:{P}_{elr}$); however, the electrolyser’s June nightly operations is challenging because of low RES power availability, which, in turn, causes it to use full grid connection power ($\:{P}_{gcm}$), i.e., 200 kW as a backup, on June nights. Moreover, the ESM charging/discharging power ($\:{P}_{esm,ch}/{P}_{esm,dch}$) shows its activity at night in January due to available wind spillage and the average $\:{SOC}_{esm}$ is around 40% (Fig. 11(c)), but the ESM is almost completely inactive at night in June (Fig. 11(d)). In both months, there is a peak in the ESM charging power profile in the first half of the daytime and another one in the ESM discharging power profile in the second half of the daytime. Both peaks are due to the solar spillage, where they are larger in June. In the first half of the daytime, the ESM is empty to store the solar spillage and in the second half, it has been charged and ready to cover the lack of RES power. Due to the battery size and the power demand, discharging the ESM does not take long at night.

### Search space and boundary plans

To generate the search space for the sizing problem, the installed power of solar/wind generating power module is assumed to be limited from 0 MWp to 3 MWp by 0.5 MW steps, the LFP battery capacity is assumed as 1 MWh to 10 MWh by 1 MWh steps, and the grid connection rated power can be 0 kW for only renewable plans (WOGC), 200 kW (GC200), or 500 kW (GC500). For each combination of these values, i.e., each plan, the technical long-term model is simulated using Editor and Simulink environments in MATLAB with a 10-minute sample time for one year. The project lifetime is assumed to be 25 years to calculate LCOH. The search space consists of 1470 different combinations/plans, which makes it difficult to show all metrics for all these plans.

**Fig. 12:**
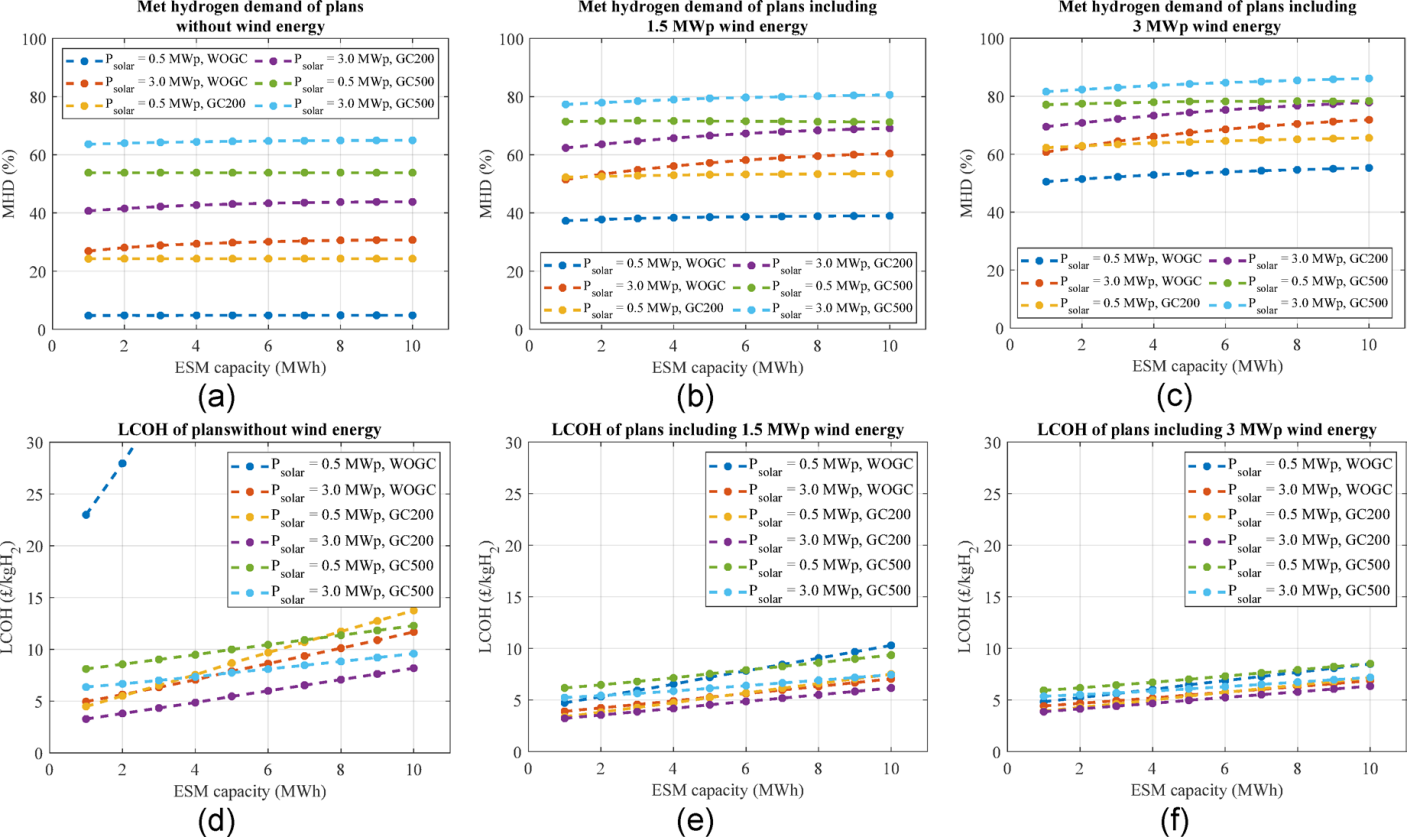
A part of search space showing the MHD of corresponding plans including a wind peak power of **a** 0 MWp, **b** 1.5 MWp, and **c** 3 MWp, and CAPEX including a wind peak power of **d** 0 MWp, **e** 1.5 MWp, and **f** 3 MWp.

Figure 12 shows MHD and LCOH only for boundary plans. The maximum MHD obtained for WOGC, GC200, and GC500 is 72%, 78%, and 86%, respectively, which is for the maximum renewable power and ESM capacity, i.e., 6 MWp and 10 MWh (See Fig. 12(c)). It is obvious that reaching higher MHD values especially 100%, which was the initial request of the stakeholder, is not possible considering the required sizes of renewables and the ESM. Furthermore, most graphs do not show a considerable increase in MHD by increasing the ESM size. Since the CAPEX is highly affected by the ESM size not shown here, e.g., from £4.4 m to £9.6 m for plans including 0.5 MWp and 3 MWp, respectively and both having 3 MWp wind power without a grid connection, the LCOH is strongly increased by increasing the ESM capacity. Another important point is the grid effect on high-RES power plans. Although GC500 increased the MHD by around 14–20% compared to only renewable plans, the LCOE is not improved. This means that the grid connection has not provided an affordable solution even as a large-size backup.

### Results for the proposed multi-metric sizing method (unconstrained metrics)

The proposed optimisation method in the section "Proposed methodology for sizing problem" is used to find the best plans according to the proposed metrics. The weighting coefficient vector is suggested as [0.2 0.1 0.04 0.04 0.04 0.18 0.2 0.2] using a simple AHP method and assuming the highest importance (0.2) for MHD, CAPEX, and LCOH, the lowest importance (0.04) for RES installed power efficiency, ESM installed capacity efficiency and SOH, and a medium importance (0.1 and 0.18) for the RES energy use efficiency and GCS, respectively. Table 6 under Scenario 1 (unconstrained metrics) shows the results when no metric is constrained. The best plan, considering the proposed metrics using the above-mentioned coefficient vector in overall the search space, is the only RES plan, which is called Plan 1. The Best GC200 plan is called Plan 2, and the best GC500 plan happens after the other two, which is called Plan 3 in Table 6. For all three plans, 1–1.5.5 MWp wind power, 2–2.5.5 MWp solar power, and 1 MWh LFP battery capacity are required, i.e., 3–4 MWp mixed RES and 1 MWh LFP battery.

The MHD increases from Plan 1 to Plan 3 because of employing a grid connection, increasing its rated power, and installing more RES. In terms of CAPEX, the best of the three plans is Plan 1 having £2.3 m without a grid connection, but from an LCOH viewpoint, Plan 2 is the best having £3.2/kgH_2_. Although the RES installed power efficiency is slightly improved from Plan 1 to Plan 3, the RES energy use efficiency shows a reverse relation by being reduced from 94.1% to 83.9%. These different changes are due to the effect of more installed RES in Plan 2 and Plan 3 on more hydrogen generation from one side affecting the RES installed power efficiency, and more spilling power affecting the RES energy use efficiency from another side. Other metrics like SOH and GCS can be compared using Table 6.

**Table 6:** Best power plant plans using the proposed optimisation method considering no limited metrics.

	S1 (Unconstrained metrics)			S2 (MHD > 60%)			S3 (MHD > 60% & GCS < 20%)
Plan name	Plan 1	Plan 2	Plan 3	Plan 4	Plan 5	Plan 6	Plan 7
GC rated power (MW)	0	0.2	0.5	0.2	0	0.5	0.2
Wind power (MWp)	1	1.5	1.5	1.5	3	1.5	1.5
Solar power (MWp)	2	2	2.5	2.5	3	3	3
Battery capacity (MWh)	1	1	1	1	1	1	1
MHD (%)	41	60	76.5	61	61	77.3	62.3
RES energy use efficiency (%)	94.1	87.7	83.9	83.9	61.7	80	80
RES installed power efficiency (kgH_2_/y/kWp)	19.5	24.4	27.2	21.8	14.4	24.5	19.7
ESM installed capacity efficiency (kgH_2_/y/kWh-ESM)	58.6	85.4	109.0	87.4	85.6	110.2	88.9
ESM SOH (%)	90.1	89.1	87.8	88.5	88.1	87.4	88
GCS (%)	0	22.2	39.4	20.6	0	37.5	19.4
CAPEX (£m)	2.3	2.9	3.1	3.1	5	3.2	3.2
LCOH (£/kgH_2_)	4.1	3.2	5.3	3.2	4.4	5.2	3.2

### Results for the proposed multi-metric sizing method (constrained metrics)

The proposed multi-metric sizing method can be constrained to achieve specific metric values, e.g., to consider a minimum acceptable value for MHD. Assuming the stakeholder needs a minimum MHD of 60%, the results are shown under Scenario 2 (MHD > 60%) in Table 6. The best plan in the overall search space according to by the proposed constrained optimisation method is a GC200 plan called Plan 4. Then, the best only-RES plan called Plan 5 and the best GC500 plan called Plan 6 are considered as the best plan of their groups, i.e., the only-RES and GC500, respectively. The total RES installed power in these three plans is 4 MWp, 6 MWp, and 4.5 MWp, respectively, which are at the high band of the RES installed power. The suggested LFP battery capacity is 1 MWh for all plans, which shows that the main challenge of the sizing problem for the hydrogen generation system is limited sources of energy, and thus a higher battery capacity is not appropriate.

Since the MHD is the same for Plan 4 and Plan 5, which is 61%, they can be compared via other metrics. The CAPEX and LCOH of Plan 4 are remarkably less than Plan 5’s, i.e., £3.1 m and £3.2/kgH_2_ against £5 m and £4.4/kgH_2_. All efficiency values of Plan 4 are more than Plan 5’s. The ESM SOH is approximately the same in both plans. The grid share of MHD for Plan 4 is only 20.6%, which means about 80% of MHD is provided by the RES. Therefore, using the grid connection as a complementary and backup source in Plan 4 strongly seems to be reasonable. Plan 6 having 77.3% MHD but 37.5% grid share of MHD is available for the stakeholder decision-making to be rejected due to the high grid share of MHD and the corresponding greenhouse gas emissions or to be accepted because of its high MHD. Note that the grid connection energy can be purchased based on renewable energy guarantees of origin (REGO) scheme to avoid/minimise carbon footprint^49^.

Another constrained optimisation problem can be solved assuming GCS < 20% in addition to MHD > 60% to avoid high-GCS plans. The results for this scenario do not include any plan from GC500. The best only-RES plan is Plan 5 and the best GC200 plan called Plan 7 is a plan like Plan 4 except a more 0.5 MWp solar power. Plan 7’s sizes and metrics are shown in the last column of Table 6. Comparing Plan 2, Plan 4, and Plan 7, which all have a 200 kW grid connection, 1.5 MWp wind power, and 1 MWh LFP battery capacity, which are different only in the solar installed power, one can deduce Plan 4 with 2.5 MWp solar power has roughly better metrics in total.

### Seasonal changes through monthly energy profiles

**Fig. 13:**
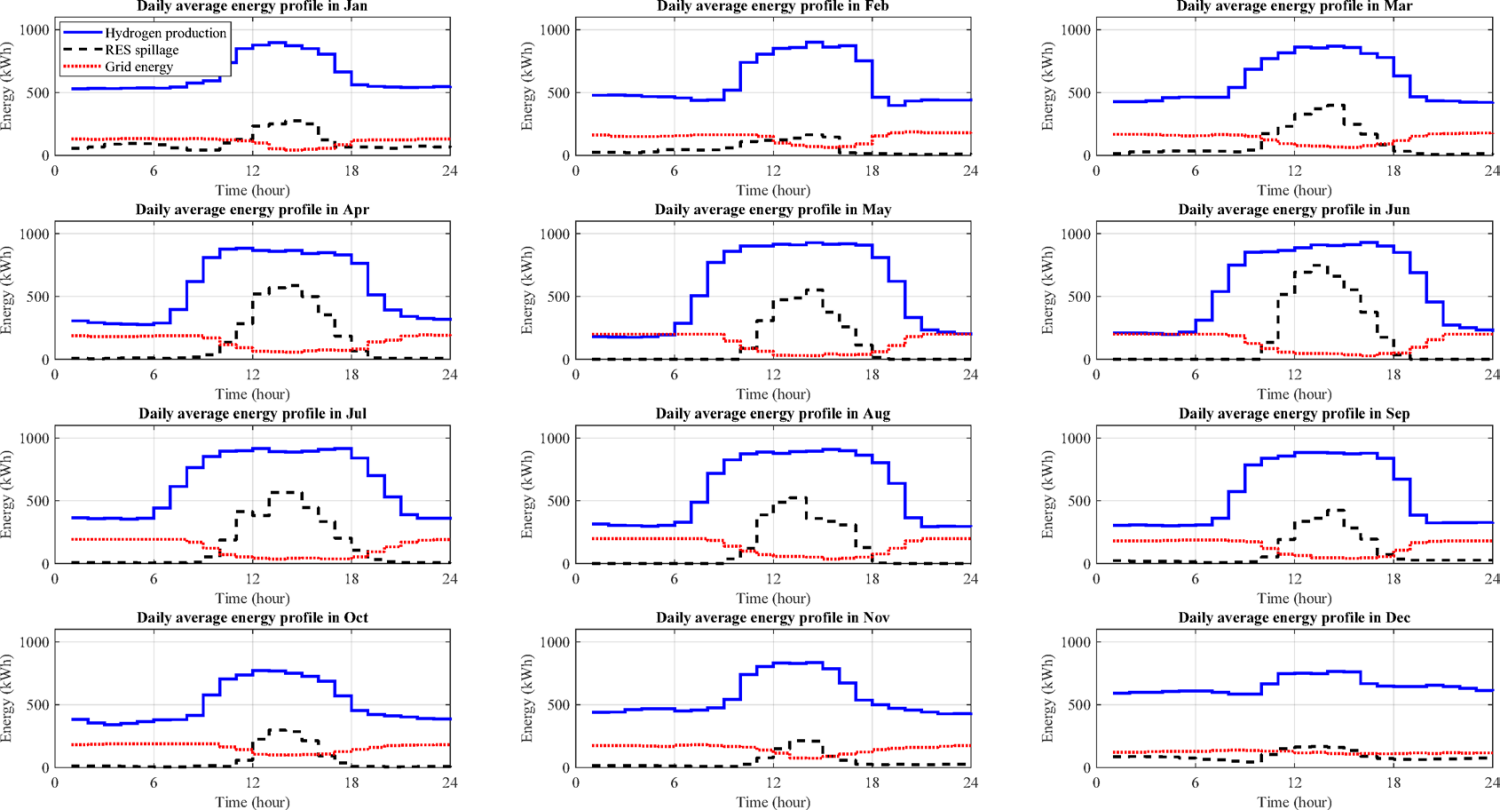
Monthly separated daily average energy profiles of Plan 4 including the required energy for the hydrogen production (solid blue), the RES spillage (dashed black), and the grid energy (dotted red).

This section is to show details of corresponding energy profiles to three important metrics including the MHD, RES energy efficiency, and GCS for the first year of the studied system operation. Figure 13 shows monthly separated daily average energy profiles of Plan 4 including the required energy for the hydrogen production (solid blue), the RES spillage (dashed black), and the grid energy (dotted red). The required energy for the hydrogen production is calculated by integrating $\:{\dot{m}}_{hgm}\left(t\right)$ to find the total first-year hydrogen production and then dividing by the average of the first-year electrolyser efficiency to calculate the equivalent electrolyser consumed electrical power. Finally, each one of these energy profiles is obtained by averaging all daily power profiles in each month. Note that energy values are calculated hourly and are not accumulative energy values.

The energy profiles equivalent to the hydrogen productions show a minimum of 500 kWh in most hours in the first and last trimesters demonstrating more available wind energy in these months. On the other hand, more daytime generation close to the maximum electrolyser demand, i.e., 1000 kW, in the other six months from April to September is obvious, which shows the higher solar generation in these months. In these six months, the grid must participate in night-time hydrogen generation more than the first and last trimesters. Finally, a considerable RES spillage especially in the middle two trimesters can be seen, which neither can be used directly nor can be stored in the 1 MWh LFP battery. As Table 6 shows and discussed earlier the RES efficiency improvement of more than a high value like 83.9% for Plan 4 is not affordable and not easily doable without affecting other metrics, e.g., increasing CAPEX and LCOH. Therefore, to make the overall project much more efficient regarding RES usage, the spillage should be used for other aims like heating and air conditioning, which is worth for future work.

### Sensitivity analysis on input weather data

**Fig. 14:**
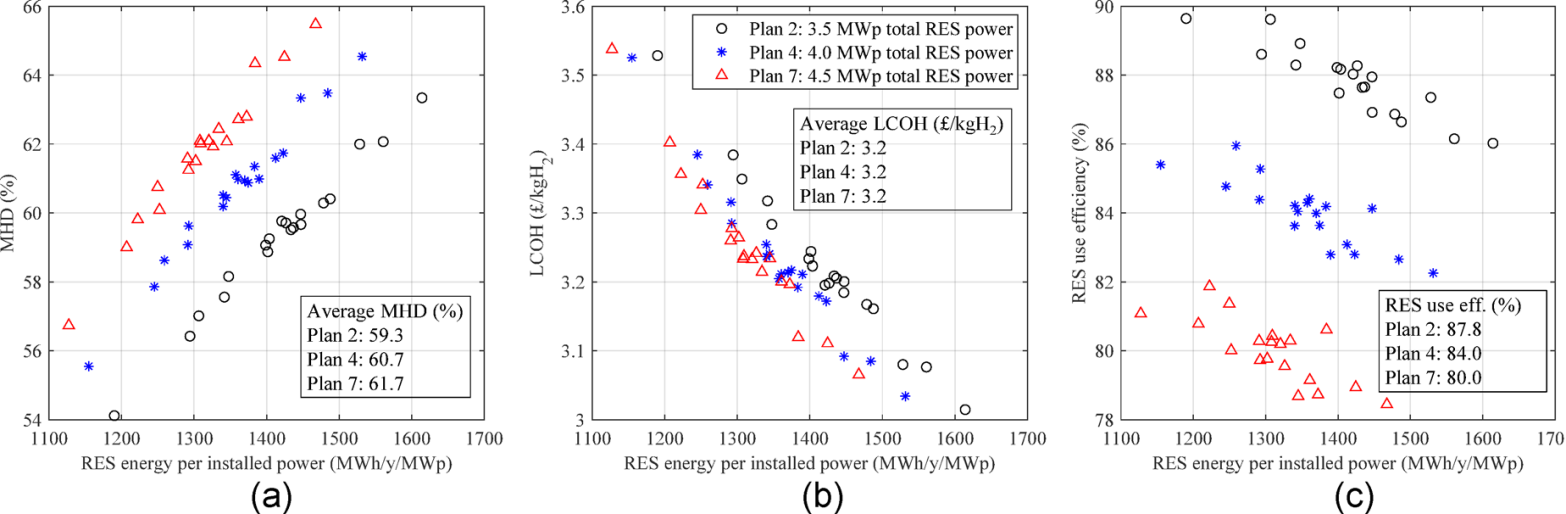
Impact of different weather situations for years 2005–2023 on **a** MHD, **b** LCOH, and **c** RES use efficiency for Plans 2, 4, and 7 all having 1.5 MWp wind power, 1 MWh battery, and a 200 kW grid connection.

Because of limitations of acceptable weather data, as well as to lower the calculation burden, the metrics used in the optimal sizing were obtained for a specific year, 2023. However, a sensitivity analysis can show the impact of different annual weather conditions on the most important metrics, which is done using the 19 years of available hourly data (2005–2023) from the PVGIS database. Figure 14(a) shows the sensitivity analysis of MHD for Plan 2, Plan 4, and Plan 7, which all have 1.5 MWp wind power, 1 MWh battery, and a 200 kW grid connection. These plans are only different in their installed solar power, leading to total RES installation of 3.5 MWp, 4 MWp, and 4.5 MWp, respectively. Instead of showing the years on the horizontal axis for the obtained MHD values, the corresponding annual available RES energy per installed power ratio in MWh/y/MWp is shown. For all three plans, the MHD increases when the ratio increases. In terms of the selected Plan 4, the MHD fluctuates between 55.8% and 64.5% with the average of 60.7% calculated from all 19 values. This is very close to the obtained MHD, 61%, for the base case scenario of Plan 4 in Table 6. However, the MHD of the worst case is 55.7% for the minimum ratio as 1160 MWh/y/MWp. Similar statistics and behaviours can be seen for Plan 2 and Plan 7. Figure 14(b) shows the LCOH for the same sensitivity analysis. The LCOH generally decreases for the three plans by increasing the RES energy per installed power ratio. It changes in a narrow band from £3/kgH_2_ to £3.6/kgH_2_, and its average is £3.2/kgH_2_ for each plan by averaging all 19 values. The LCOH is also considerably robust against the RES generation uncertainties. Finally, Fig. 14(c) shows the RES use efficiency changes against the RES energy per installed power ratio, which is about 4% for each plan and with the same decreasing behaviour as the LCOH. The average (band) of RES use efficiency increases (shifts up) from Plan 7 to Plan 4 and then to Plan 2. It is because of increasing spillage in plans with a higher installed power.

### Sensitivity analysis on economic parameters

**Fig. 15:**
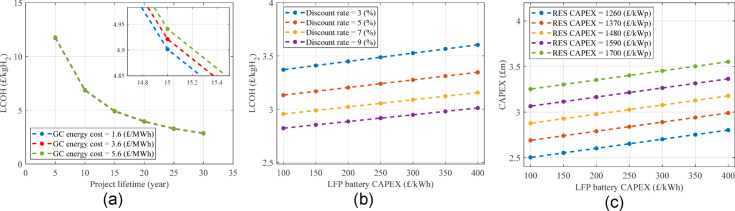
Impact of economic parameters on the LCOH and CAPEX of Plan 4: **a** the grid energy cost and project lifetime impact on the LCOH, **b** the discount rate and battery unit CAPEX impact on the LCOH, and **c** the RES unit CAPEX and battery unit CAPEX impact on the total CAPEX.

Another sensitivity analysis is performed to investigate the impact of important economic parameters on the LCOH and CAPEX of the selected Plan 4. Figure 15(a) shows the grid energy cost and project lifetime impact on the LCOH. The grid energy cost in the base case is £1.6/MWh, which is the energy-related term of the grid costs and was assumed to be from non-renewable energy. To investigate the impact of purchasing renewable energy from the grid based on REGO agreements, only this term is affected. The other three terms of grid costs shown in Table 5 are not dependent on the energy type provided for the HGM. The LCOH is slightly affected by increasing the grid energy cost to £3.6/MWh and even £5.6/MWh, which are reasonable prices for renewable energy purchasing through REGO agreements. The second important parameter in LCOH calculation is the project lifetime, which is usually 25 or 30 years for hybrid energy systems, including solar and wind systems. Figure 15(a) shows a high sensitivity of LCOH to the project lifetime, especially when it is supposed to be less than 20 years. In this range of lifetime, the LCOH is remarkably high, and the project may not be affordable.

Figure 15(b) shows the impact of the discount rate and battery unit CAPEX on the LCOH. The discount rate is strongly affected by various economic and non-economic factors and may change over the years. It was assumed as 5% for the base case studies, and it changes between 3% and 9% in this sensitivity analysis. Increasing the discount rate leads to a relatively remarkable decrease in the LCOH. On the other hand, battery storage technologies have been extremely developed in recent years, resulting in a considerable decrease in the purchasing costs. A further decrease is also expected for future years^50^. The sensitivity analysis shown in Fig. 15(b) shows a decrease in the LCOH of about £0.2/kgH_2_ for each value of discount rate when the LFP battery unit CAPEX decreases from £400/kWh to £100/kWh, which shows a high robustness of the LCOH of the selected plan against the LFP battery purchasing and replacement costs.

In the third sensitivity analysis, the total CAPEX of the selected Plan 4 is analysed against the LFP battery and RES unit CAPEX changes in their reasonable ranges, which is shown in Fig. 15(c). The CAPEX decreases by about £0.3 m when the LFP battery unit CAPEX decreases from £400/kWh to £100/kWh. The RES unit CAPEX is the sum of the solar and wind CAPEX terms. These per-unit cost terms are provided in Table 5 for the base case, namely pre-development and construction costs under both solar and wind cost lists. For the base case scenario, the RES unit CAPEX is £1480/kWp (= 50 + 200 + 130 + 1100). To find the range of RES CAPEX shown in Fig. 15(c), the pre-development cost term including the technology cost is affected by a multiplier to the base case value with a change of 0.25 per each step and the construction cost is affected by a multiplier to the base case value with a change of 0.05 per each step to follow reasonable RES cost decrease patterns over future years reported in^50^. The total CAPEX diminishes about £0.2 m for any step change in the RES unit CAPEX. Assuming the total LFP battery and RES unit CAPEX decrease is limited to one step during the decision-making period in the worst case, i.e., £0.5 m, uncertainties of these important parameters may have a considerable effect on the total CAPEX as 16% (= 100$\:\times\:$0.5/3.1), which needs to be considered.

## Conclusion

A modelling method is proposed for long-term investigations of renewable-based power plants supplying hydrogen generation and storage stations, including both electrical power generation and demand sides, which generally consists of solar and wind energy resources, energy storage systems, backup resources, and hydrogen system modules. The model, which can be generalised to many case studies because of its modular structure, is reinforced by proposing a comprehensive set of metrics to be used in sizing the power plant through a multi-objective optimisation function. The power plant designer can study different aspects of each design/plan and make a precise decision using the proposed modelling and sizing methods. A set of primary modelling method outputs in the form of different power and battery state of charge profiles validated reasonable behaviours of the proposed long-term model of a case study. Moreover, the met hydrogen demand and levelised cost of hydrogen were shown for a range of boundary plans in the search space of the optimisation problem, which can be considered as the secondary outputs of the model to validate its accuracy because of their reasonable behaviours against the sizes of the main power plant modules. Although the multi-metric optimisation method results depend on weighting coefficients, selecting a sensible set of coefficients according to their importance level for the designer/stakeholder can easily be done. Therefore, several plans can be found so that each one is the best plan based on the corresponding set of coefficients, i.e., weighted metrics.

For the case study to maximise production of a hydrogen system including a 1 MW electrolyser, the most affordable plan that can only meet 61% of the hydrogen full-time demand consists of 1.5 MWp installed wind power, 2.5 MWp installed solar power, 1 MWh LFP battery capacity and a 200 kW grid connection as the backup. The capital expenses in the first year and the levelised cost of hydrogen calculated in 25 years are £3.1 m and £3.2/kgH_2_ for this plan. This plan can limit the grid share of the total energy required for producing hydrogen in one year to 20.6% and can achieve 83.9% renewable energy use efficiency, i.e., 15.9% renewable energy spillage. The monthly energy profiles showed that the majority of renewable energy spillage happens in the summer months due to excess solar energy. The sensitivity analysis results show the impact of uncertainties of weather data and economic parameters on the most important metrics defined for the system, which need to be considered in the decision-making process.

As future work, one can study a more efficient operation of these hybrid energy systems by modelling and considering other types of energy consumption, like heating/cooling systems, to improve renewable energy use efficiency. Moreover, the proposed model can be enhanced for case studies with more detailed technical information on operation and control, such as voltage and frequency regulation, to investigate their impact on optimised sizes.

## Data Availability

The data will be available on reasonable request by contacting Mobin Naderi ([m.naderi@sheffield.ac.uk](mailto: m.naderi@sheffield.ac.uk)).

## References

[1] Hydrogen as a low emission fuel. (accessed 22 Oct 2025). https://www.iea.org/energy-system/low-emissions-fuels/hydrogen (2025).

[2] Energy density for different materials. (accessed 22 Oct 2025). https://chemistry.beloit.edu/edetc/SlideShow/slides/energy/density.html (2025).

[3] Hydrogen strategy update to the market. (accessed 02 Oct 2025). https://www.gov.uk/government/publications/hydrogen-strategy-update-to-the-market-december-2024?utm_source=chatgpt.com (2024).

[4] Hydrogen infrastructure requirements up to 2035. (accessed 02 Oct 2025). https://www.gov.uk/government/publications/hydrogen-infrastructure-requirements-up-to-2035?utm_source=chatgpt.com (2022).

[5] Ge, L. et al. A review of hydrogen generation, storage, and applications in power system. *J. Energy Storage*. **75**, 109307. 10.1016/j.est.2023.109307 (2024).

[6] Togun, H. et al. A review on recent advances on improving fuel economy and performance of a fuel cell hybrid electric vehicle. *Int. J. Hydrogen Energy*. **89**, 22–47. 10.1016/j.ijhydene.2024.09.298 (2024).

[7] Hawkes, R. GB renewables map. (accessed 22 Oct 2025). https://renewables-map.robinhawkes.com/curtailment.

[8] Chavez, D. L., Azzaro-Pantel, C., Montignac, F. & Ruby, A. Integrating life cycle assessment in multi-objective optimization of green hydrogen systems: a review of literature and methodological challenges. *Renew. Sustain. Energy Reviews*. **217**, 115689–115706. 10.1016/j.rser.2025.115689 (2025).

[9] Ansari, A. B. Multi-objective size optimization and economic analysis of a hydrogen-based standalone hybrid energy system for a health care center. *Int. J. Hydrogen Energy*. **62**, 1154–1170. 10.1016/j.ijhydene.2024.03.165 (2024).

[10] Ríos, C., Molina, P., de León, C. M. & Brey, J. J. Simulation of the optimal plant size to produce renewable hydrogen based on the available electricity. *Int. J. Hydrogen Energy*. **52**, 1325–1337. 10.1016/j.ijhydene.2023.08.306 (2024).

[11] Gökçek, M. et al. Optimum sizing of hybrid renewable power systems for on-site hydrogen refuelling stations: case studies from Türkiye and Spain. *Int. J. Hydrog. Energy* **59**, 715–29. 10.1016/j.ijhydene.2024.02.068 (2024).

[12] Palanisamy, S. & Lala, H. Optimal sizing of renewable energy powered hydrogen and electric vehicle charging station (HEVCS). *IEEE Access.* **12**, 48239–48254. 10.1109/ACCESS.2024.3383960 (2024).

[13] Okonkwo, P. C., Nwokolo, S. C., Meyer, E. L., Ahia, C. C. & Mansir, I. B. Techno-economic optimization of renewable hydrogen infrastructure via AI-based dynamic pricing. *Sci. Rep.* **15**, 31529–31561 (2025). https://www.nature.com/articles/s41598-025-17506-z 40866504 10.1038/s41598-025-17506-z PMC12391492

[14] Lebepe, M. C., Oviroh, P. O. & Jen, T. C. Techno-economic optimisation modelling of a solar-powered hydrogen production system for green hydrogen generation. Sustain. *Energy Res.* **11**, 1–20. 10.1186/s40807-025-00151-5 (2025).

[15] Sayed, K. et al. Feasibility study and economic analysis of PV/wind-powered hydrogen production plant. *IEEE Access.* **12**, 76304–76318. 10.1109/ACCESS.2024.3406895 (2024).

[16] Marocco, P., Gandiglio, M., Cianella, R., Capra, M. & Santarelli, M. Design of hydrogen production systems powered by solar and wind energy: an insight into the optimal size ratios. *Energy Convers. Manag*. **15**, 314:118646. 10.1016/j.enconman.2024.118646 (2024).

[17] Fabianek, P. & Madlener, R. Techno-economic analysis and optimal sizing of hybrid PV-wind systems for hydrogen production by PEM electrolysis in California and Northern Germany. *Int. J. Hydrogen Energy*. **20**, 67:1157–1172. 10.1016/j.ijhydene.2023.11.196 (2024).

[18] Oyewole, O. L., Nwulu, N. I. & Okampo, E. J. Optimal design of hydrogen-based storage with a hybrid renewable energy system considering economic and environmental uncertainties. *Energy Convers. Manag*. **300**, 117991. 10.1016/j.enconman.2023.117991 (2024).

[19] Mkhaitari, R., Yamina, M., Zazoui, M. & Elrhezouani, F. Technical sizing of renewable energy capacity for large-scale green hydrogen production. *Energy Sustain. Dev.* **84**, 101595–101602. 10.1016/j.esd.2024.101595 (2025).

[20] Jovan, D. J., Pregelj, B., Sekavčnik, M. & Dolanc, G. Sizing of a hydrogen system for green-hydrogen production by utilising surplus water accumulation in a hydropower plant. *Renew. Energy*. **255**, 123849–123864. 10.1016/j.renene.2025.123849 (2025).

[21] Franco, A., Carcasci, C., Ademollo, A., Calabrese, M. & Giovannini, C. Integrated plant design for green hydrogen production and power generation in photovoltaic systems: balancing electrolyzer sizing and storage. *Hydrogen* **6**, 7–28. 10.3390/hydrogen6010007 (2025).

[22] Al-Mahmodi, M., Ayadi, O., Wang, Y. & Al-Halhouli, A. Sensitivity-based techno-economic assessment approach for electrolyzer integration with hybrid photovoltaic-wind plants for green hydrogen production. *Int. J. Hydrogen Energy*. **97**, 904–919. 10.1016/j.ijhydene.2024.12.002 (2025).

[23] Thakkar, N. Sensitivity analysis of reliability constrained, eco optimal solar, wind, hydrogen storage based islanded power system. *Sci. Rep.* **15**, 9743–9758 (2025). https://www.nature.com/articles/s41598-025-92893-x 40118950 10.1038/s41598-025-92893-x PMC11928496

[24] Abirami, M., Hariprasath, D. & Vighneshwari & Techno-economic optimization of hybrid renewable systems for sustainable energy solutions. *Sci. Rep.* **15**, 1:1–30 (2025). https://www.nature.com/articles/s41598-025-08171-3 40596586 10.1038/s41598-025-08171-3 PMC12219102

[25] Grid-Connected Battery Energy Storage System (BESS). Growth Statistics. (accessed 22 Oct 2025). https://www.vertexmarketresearch.com/reports/grid-connected-battery-energy-storage-market (2025).

[26] Photovoltaic geographical information system. (accessed 22 Oct 2025). https://re.jrc.ec.europa.eu/pvg_tools/en/#api_5.3 (2025).

[27] Naderi, M. et al. Techno-economic planning of a fully renewable energy-based autonomous microgrid with both single and hybrid energy storage systems. *Energies* **17**, 788. 10.3390/en17040788 (2024).

[28] Simões, F., Henriques, C., Figueiredo, N. C. & da Silva, P. P. Efficient power purchase agreement structures for meeting corporate electricity needs with solar energy. *Energy*10.1016/j.energy.2025.135651 (2025).

[29] Introduction to power purchase agreement. Crown commercial service. (accessed 18 Dec 2025). https://assets.crowncommercial.gov.uk/wp-content/uploads/Power-Purchase-Agreements-PPA-An-Introduction-to-PPAs.pdf (2020).

[30] Reddi, K., Elgowainy, A., Rustagi, N. & Gupta, E. Two-tier pressure consolidation operation method for hydrogen refueling station cost reduction. *Int. J. Hydrogen Energy*. **43**, 2919–2929. 10.1016/j.ijhydene.2017.12.125 (2018).

[31] Gresham House Energy Storage Fund. Capacity augmentations the priority for 2025. (accessed 22 Oct 2025). https://www.edisongroup.com/research/capacity-augmentations-the-priority-for-2025/34068/?utm_source=chatgpt.com (2025).

[32] IEC 62933-2-1. General requirements for grid-integrated energy storage systems. International Electrotechnical Commission. (accessed 22 Oct 2025). https://webstore.iec.ch/en/publication/27124 (2017).

[33] IEEE 1679.1. Guide for the characterization and evaluation of lithium-based batteries in ESS. IEEE Standards Association. (accessed 22 Oct 2025). https://standards.ieee.org/ieee/1679.1/7222/ (2017).

[34] Deb, K. Multi-objective optimisation using evolutionary algorithms: an introduction. In *Multi-objective Evolutionary Optimisation for Product Design and Manufacturing*, 3–34 (Springer, 2011). 10.1007/978-0-85729-652-8_1

[35] Gunantara, N. A review of multi-objective optimization: Methods and its applications. *Cogent Eng.*10.1080/23311916.2018.1502242 (2018).

[36] Marler, R. T. & Arora, J. S. Survey of multi-objective optimization methods for engineering. *Struct. Multidisciplinary Optim.* **26**, 369–395. 10.1007/s00158-003-0368-6 (2004). https://link.springer.com/article/

[37] ITM power report. (accessed 22 Oct 2025). https://northsearegion.eu/media/9385/feasibility-of-hydrogen-bunkering-final-080419.pdf (2019).

[38] D-type diaphragm. compressor model GD4-250/10–300. (accessed 22 Oct 2025). https://www.sollant.com/diaphragm-compressor/ (2025).

[39] JA Solar 595 W N-type Bifacial Double Glass Solar Panel. (accessed 22 Oct 2025). https://www.pluginsolar.co.uk/?product=ja-solar-595w-n-type-bifacial-double-glass-half-cell-mbb-solar-panel-traceable#tab-description (2025).

[40] Riva Calzoni, A. T. B. ATB 500.54. (accessed 22 Oct 2025). https://en.wind-turbine-models.com/turbines/1533-atb-riva-calzoni-atb-500.54 (2025).

[41] Lewerenz, M. et al. Systematic aging of commercial LiFePO4| Graphite cylindrical cells including a theory explaining rise of capacity during aging. *J. Power Sources* **345**, 254–63. 10.1016/j.jpowsour.2017.01.133 (2017).

[42] Krupp, A. et al. Calendar aging model for lithium-ion batteries considering the influence of cell characterization. *J. Energy Storage*. **45**, 103506. 10.1016/j.est.2021.103506 (2022).

[43] Electrolyser cost, European Hydrogen Observatory. (accessed 22 Oct 2025). https://observatory.clean-hydrogen.europa.eu/hydrogen-landscape/production-trade-and-cost/electrolyser-cost?utm_source=chatgpt.com (2024).

[44] Electricity Generation Costs DESNZ report. (accessed 22 Oct 2025). https://assets.publishing.service.gov.uk/media/6556027d046ed400148b99fe/electricity-generation-costs-2023.pdf?utm_source=chatgpt.com (2023).

[45] Real Cost Behind Grid-Scale Battery Storage. (accessed 22 Oct 2025). https://www.euro-inox.org/real-cost-behind-grid-scale-battery-storage-2024-european-market-analysis/?utm_source=chatgpt.com (2025).

[46] Fu, R., Remo, T. & Margolis, R. U. S. Utility-Scale Photovoltaics Plus-Energy Storage System Costs Benchmark, National Renewable Energy Laboratory. (accessed 22 Oct 2025). https://www.nrel.gov/docs/fy19osti/71714.pdf (2018).

[47] Connections Charging Statements and Methodology. (accessed 22 Oct 2025). https://connections.nationalgrid.co.uk/connections-charging-statements/ (2025).

[48] Quarterly Energy Prices. March 2025. (accessed 22 Oct 2025). https://www.gov.uk/government/collections/quarterly-energy-prices?utm_source=chatgpt.com#2025 (2025).

[49] Renewable Energy Guarantees of Origin (REGO) scheme. Renewable Energy Guarantees of Origin Guidance for generators, agents and suppliers | Ofgem. (accessed 22 Oct 2025) (2023).

[50] Battery energy storage. cost analysis, Annual technology baseline, National Laboratory of the Rockies (accessed 18 Dec 2025). https://atb.nrel.gov/electricity/2024/commercial_battery_storage (2024).

